# Which explainable AI methods in medical imaging are clinically impactful? A systematic literature review addressing the clinician's perspective

**DOI:** 10.3389/frai.2026.1819422

**Published:** 2026-05-29

**Authors:** Shahab Ud Din, Ruby Kemna, Johannes C. F. Ket, Muhammad Iqbal, Omar Bohoudi, Mark Hoogendoorn, Elena Beretta, Aneta Lisowska

**Affiliations:** 1 Department of Computer Science, Vrije Universiteit, Amsterdam, Netherlands; 2 Department of Surgery, Amsterdam UMC, Amsterdam, Netherlands; 3 Cancer Center Amsterdam, Amsterdam UMC, Amsterdam, Netherlands; 4 Medical Library, Vrije Universiteit, Amsterdam, Netherlands; 5 Computer and Information Science, Higher Colleges of Technology, Fujairah, United Arab Emirates; 6 Department of Radiation Oncology, Amsterdam UMC, Amsterdam, Netherlands

**Keywords:** clinician-centered evaluation, explainability, explainable artificial intelligence, human-AI collaboration, interpretability, machine learning, medical imaging, trust

## Abstract

**Background:**

Explainable Artificial Intelligence (XAI) has emerged as a strategy to enhance the transparency and interpretability of AI systems in medical imaging. Although numerous methods have been developed to generate explanations of model behavior, their evaluation has predominantly relied on technical performance metrics rather than clinician-centered assessment. The limited involvement of clinicians in the development and validation of XAI methods, together with the absence of clinically meaningful evaluation frameworks, represents a significant barrier to the successful integration of AI into routine clinical workflows.

**Objective:**

To conduct a comprehensive review of the existing literature on the clinician-centered evaluation of XAI techniques in the domain of medical imaging.

**Method:**

This systematic review was conducted in accordance with the Preferred Reporting Items for Systematic Reviews and Meta-Analyzes (PRISMA 2020) guidelines. A literature search (in Medline, Web of Science, IEEE, ACM Digital Library and Scopus) was performed from inception up to November 17, 2025 in collaboration with a medical information specialist. The study protocol was prospectively registered in the International Prospective Register of Systematic Reviews (PROSPERO; registration number CRD420261301196). Any modifications to the original protocol are recorded in the PROSPERO entry and are reported in this manuscript where applicable. Study designs were categorized using MMAT and risk of bias was assessed with a sample-size adjustment.

**Results:**

We identified 9,305 records from five databases, which were reduced to 5,687 after removing duplicates. Following title and abstract screening, 5,440 articles were excluded as irrelevant. Full-text assessment led to the exclusion of 192 articles, primarily because they did not involve healthcare professionals in evaluating explainability (90 studies) or were unrelated to medical imaging (73 studies). Ultimately, 51 studies met the inclusion criteria and were independently reviewed, and bibliographic details and key contributions to XAI in medical imaging were extracted.

**Conclusions:**

Clinician-centered evaluation of XAI in medical imaging is expanding but remains methodologically fragile. The available evidence suggests that the type of explanation may influence evaluation outcomes. In several studies, example-based and concept-based methods are associated with improvements in both subjective and objective measures, as well as with assessments of automation bias. In contrast, attribution-based explanations are more frequently linked to enhanced clinician perceptions, while their relationship with decision-making outcomes and automation bias remains less clear.

**Systematic review registration:**

https://www.crd.york.ac.uk/PROSPERO/view/CRD420261301196

## 1 Introduction

The integration of artificial intelligence (AI) into medical imaging has been expected to enhance diagnostic accuracy and improve clinical decision-making (Ahmed et al., 2026). In recent years, machine learning algorithms have demonstrated performance comparable to or exceeding that of human experts in detecting disease patterns and abnormalities across diverse imaging modalities, including computed tomography (CT) (Yang et al., 2025), magnetic resonance imaging (MRI) (Guo et al., 2024), X-rays (Feng et al., 2025b), and pathological images (Arunachalam et al., 2025). Despite these advances, a fundamental challenge persists: many machine learning models achieve strong predictive accuracy but rely on complex architectures that make them largely opaque, functioning as “black-box” systems with little interpretability of their decision-making processes (Bhatia et al., 2025). Successful clinical implementation of AI-driven diagnostic methods requires not only high predictive accuracy, but also requires that healthcare professionals understand and trust the model's decision-making process (Ahmed et al., 2026). Clinicians must be able to interpret and validate AI-generated predictions in the context of their clinical knowledge to make informed diagnostic and treatment decisions (Ramos-Soto et al., 2025; Hassan et al., 2024). The absence or lack of explainability remains a major obstacle to clinical adoption, particularly in high-stake medical environments where accountability, transparency, and trust are essential (Teng et al., 2024).

Explainable Artificial Intelligence (XAI) has emerged in healthcare as a response to concerns regarding the opacity of AI models, providing methods to clarify how AI systems generate diagnostic predictions (Muhammad and Bendechache, 2024). XAI techniques such as Gradient-weighted Class Activation Mapping (Grad-CAM), Local Interpretable Model-Agnostic Explanations (LIME), SHapley Additive exPlanations (SHAP), and saliency maps have been introduced to visualize and interpret the decision-making processes of machine learning models in medical imaging (Hassan et al., 2025). These approaches highlight the regions or features in medical images that most strongly influence model predictions, offering visual explanations aligned with clinical knowledge and reasoning (Ahmed et al., 2026).

Despite advances in XAI, significant gaps persist between XAI development and clinical practice. The field continues to face challenges including the standardization of explainability evaluation metrics, the integration of XAI systems into existing clinical workflows, the management of model bias and data quality issues, and the alignment of technical explanations with clinical requirements and user expectations (Mienye et al., 2024). Moreover, while numerous XAI studies demonstrate technical feasibility in experimental settings, real-world clinical adoption or implementation of XAI remains limited, in part due to insufficient engagement with clinicians during development and evaluation (Ihongbe et al., 2024). A critical distinction exists between technical validation and clinical evaluation of XAI: AI experts often prioritize transparency and traceability, whereas clinicians prioritize explainability that supports decision-making and ensures patient safety (Mienye et al., 2024). This divergence creates gaps in understanding what constitutes meaningful explainability from a clinical perspective. Clinicians require explanations that are not only technically valid but also trustworthy, clinically relevant, actionable, and aligned with established medical knowledge and diagnostic standards (Mienye et al., 2024; Ihongbe et al., 2024).

Recent systematic reviews and surveys have highlighted the rapid expansion of XAI techniques in medical imaging across a range of modalities and clinical applications, including radiology, pathology, ophthalmology, and oncology (Ahmed et al., 2026). Some of existing reviews of XAI have focused mainly on technical approaches, performance measures, and improvements in model accuracy. However, they do not address how clinicians evaluate the explanations produced by machine learning models (Ihongbe et al., 2024; Pičulin et al., 2025). A summary of recent literature reviews on explainable artificial intelligence (XAI) in medical imaging is provided in Supplementary material S1.

A notable study (Bharati et al., 2023b) reviews the application of XAI in the context of the Internet of Health Things (IoHT), but it does not address the broader medical imaging domain. Existing reviews in medical imaging (Salih et al., 2023; De Vries et al., 2023; Martin et al., 2023; Fuhrman et al., 2022; Gulum et al., 2021) are often limited in scope, focusing on specific diseases or imaging modalities. Others (Champendal et al., 2023; Bhati et al., 2024; Patŕıcio et al., 2023; Ennab and Mcheick, 2024; Van der Velden et al., 2022; Yang et al., 2022; Singh et al., 2020a; Chaddad et al., 2023; Salahuddin et al., 2022; Nazir et al., 2023; Band et al., 2023; Borys et al., 2023; Bharati et al., 2023a) adopt a broader perspective across medical imaging but do not adhere to a systematic review methodology. Although a small number of systematic reviews (Muhammad and Bendechache, 2024; Ahmed et al., 2026; Groen et al., 2022; Hassan et al., 2025) considered medical imaging in a broader context, none provides a comprehensive examination of the clinician-centered evaluation of the explanations generated by XAI systems in this medical imaging.

Therefore, this review aims to bridge this gap by systematically synthesizing evidence on how clinicians perceive, evaluate, and apply XAI in medical imaging. Specifically, it assesses the current state of clinician-centered evaluation of XAI and identifies the methods and dimensions used in these clinician focused evaluations.

### 1.1 Objective

The primary objective of this systematic literature review is to synthesize and critically appraise the existing evidence on clinician-centered evaluations of XAI in medical imaging. Specifically, this review aims to examine how healthcare professionals assess explainability; which factors influence their trust in and acceptance of XAI systems; whether and to what extent explanatory components enhance clinical utility; how explainability supports clinical decision-making;, and which dimensions or aspect of explainability are evaluated. The secondary objectives are as follows:

- To synthesize evidence on clinician's evaluation of XAI in medical imaging contexts.
- To identify which explanation approaches are preferred by clinicians across different medical imaging use cases.
- To identify the key dimensions of explainability that clinicians consider essential for the effective and responsible integration of AI into medical imaging practice.

### 1.2 Research questions

The following research questions are formulated to methodically guide the systematic review.

#### 1.2.1 Primary research question

What is the current state of clinician-centered evaluation of explainable AI (XAI) methods in medical imaging?

#### 1.2.2 Secondary research question

- What categories of XAI methods have been applied in medical imaging, and how are these methods evaluated from the clinician's perspective?
- Which evaluation dimensions are applied to assess XAI methods in medical imaging?
- What evidence has been reported across the identified evaluation dimensions?
- Which XAI methods in medical imaging are preferred by clinicians or perceived as the most impactful in clinical practice?
- Which XAI approaches are reported to provide the greatest support for clinical decision-making?

### 1.3 Scope and significance

This systematic literature review focuses solely on clinician-centered evaluations of explainable AI (XAI) in medical imaging. It includes studies that directly investigate how healthcare professionals perceive, assess, use, and experience XAI systems in medical imaging. The review synthesizes the findings of quantitative, qualitative, and mixed-methods research, and analyzes which methodological approaches most effectively capture clinicians' perspectives on explainability, trust, usability and clinical utility.

The significance of this review lies in its potential to advance the development of more clinician-oriented XAI systems and to help bridge the persistent gap between technical AI innovation and real-world clinical adoption and implementation. By systematically synthesizing evidence on clinical perspectives, this review seeks to provide researchers, developers, and healthcare organizations with evidence-based guidance to design explainable AI systems that are not only technically robust but also align with clinicians' workflows, informational needs, and decision-making processes.

## 2 Methods

### 2.1 Study design

In this systematic review, eligible studies comprised quantitative, qualitative, and mixed-methods designs investigating clinicians' perceptions and evaluations of the explainability outputs of XAI systems in medical imaging.

### 2.2 Participants

Clinicians (e.g., radiologists or other medical specialists) who interacted with XAI-based medical imaging systems.

### 2.3 Interventions and comparators

Explainable AI (XAI) methods applied to medical imaging.

#### 2.3.1 Comparator

- Different XAI methods.
- AI without explanation.

#### 2.3.2 Outcomes

- Clinician-centered evaluation dimensions, including understandability, trust, decision-making, actionability, and preference.

### 2.4 Systematic review protocol

This systematic review was conducted in accordance with the PRISMA 2020 guidelines. The review protocol was prospectively registered with the International Prospective Register of Systematic Reviews (PROSPERO; registration number CRD420261301196), before initiation of the literature search.

### 2.5 Search strategy and data sources

A comprehensive literature search was performed in the databases: OVID/Medline, Clarivate Analytics/Web of Science Core Collection, IEEE/Xplore, ACM Digital Library and Elsevier/Scopus, from inception to November 17, 2025 in collaboration with a medical information specialist (JCFK). The search included controlled and free text terms for synonyms of “physicians” or “radiologists” and “explainable” and “artificial intelligence” and “surveys” or “questionnaires”. The search was performed without restrictions for methodology, date or language. The full search strategies can be found in Supplementary material S2. Duplicate articles were excluded by the medical information specialist using Endnote X20.0.1 (Clarivate), following the Amsterdam Efficient Deduplication (AED)-method (Bramer et al., 2016) and the Bramer-method Ouzzani et al. (2016). After deduplication, the remaining search results were imported into Rayyan (Johnson and Phillips, 2018), a systematic review tool.

### 2.6 Study selection and data extraction

Prior to the screening process, a structured screening protocol was developed outlining the predefined inclusion and exclusion criteria Table 1, and was shared with all reviewers. In the first stage, titles and abstracts were screened for relevance by a multiple reviewer single time, the reviewer includes (SUD, MI, EB, AL) using Rayyan.

**Table 1:** Inclusion and exclusion criteria.

Inclusion criteria	Exclusion criteria
Medical images related articles	Articles unrelated to medical imaging
At least one AI/ML technique is used	No AI/ML technique is used
At least one explainability method is used	NO explainability method is used
Clinician/Healthcare professionals are involved in Explainability evaluation	No clinician/Healthcare professionals are involved in Explainability evaluation
Primary research (including clinician surveys/questionnaires, experiments, qualitative studies, and mixed-methods studies)	Secondary research (systematic reviews, scoping reviews, narrative reviews), editorials, commentaries, opinion papers
Peer-reviewed studies published in the considered databases	Editorials, Commentary, Opinion papers, White paper, Technical report
Full text available	Full text not available
In English language	Not in English

To ensure duplicate independent screening, a subset of 86 articles was screened at the abstract level by two reviewers to assess inter-reviewer agreement. An agreement rate of 78% was achieved, with most discrepancies occurring in the “Maybe” category. Following the initial screening, all records were additionally reviewed using the AI tool Claude Sonnet 4.5 (Claude Code). Disagreements between the AI-assisted screening and human reviewers were resolved through discussion and adjudication by (SUD) To minimize the risk of excluding potentially relevant studies, all records marked as “Maybe” were retained for full-text screening. In cases where consensus could not be reached at this stage, the study was automatically selected for full-text review to ensure a comprehensive evaluation and minimize the risk of premature exclusion. Further details are provided in Supplementary material S4.

The authors were not contacted for articles whose full texts could not be retrieved through online sources. Consequently, these studies were excluded due to full-text unavailability. In the full text screening phase, 123 eligible articles were independently and thoroughly reviewed by two reviewers (SUD and AL), four articles were excluded as the full text was not avilable. The AI tool Claude Sonnet 4.5 was additionally used to screen and extract data from 120 full-text articles. All studies included by Claude were subsequently reviewed by (SUD). Furthermore, all full text decisions whether made by human reviewers and/or Claude were independently verified by a second reviewer (MI) to ensure the accuracy of inclusion and exclusion decisions. Any disagreements about study inclusion were discussed between the reviewers and resolved by consensus. Further details are provided in Supplementary material S4.

Following study selection, the included studies were independently reviewed by the researchers (SUD and AL) using a predefined extraction form. For each study, bibliographic details and key contributions to the field of explainable AI (XAI) in medical imaging were systematically extracted. The extracted variables included the type of machine learning or deep learning models employed; XAI methods and approaches applied; datasets used; imaging modalities; targeted diseases or clinical tasks; evaluation metrics; and detailed characteristics of the study design related to clinician-centered evaluation. The extracted data was then compared across reviewers, and any discrepancies or inconsistencies were resolved through detailed discussion until consensus was reached. A summary of the data extraction fields is presented in Table 2.

**Table 2:** Summary of data extraction.

Category	Information extracted
Image and Dataset	Imaging Modality, Image Dimensionality, Single/Multi-modal? Anatomical Region, Clinical Task, Target Pathology, Gold Standard
Machine Learning Model	ML Paradigm, Model Architecture, Model Characteristics, Learning Setup, Input Type, Task Type, Label Type, Output Granularity
Explainability method	XAI Category, Specific XAI Methods, Number of XAI Methods, XAI Scope (Local/Global/Both), Intrinsic or Post-hoc, Model-agnostic or Model-specific, Explanation Output Format, Explanation Timing (Inference/Retrospective)
Clinician's evaluation	Clinical Specialty, Number of Clinicians, Years of Clinical Experience, AI Experience Level, Evaluation Type, Comparator Used, Number of Evaluation Experiments, Case Type Used, Understandability Assessed (Yes/No), Trust Assessed (Yes/No), Decision Impact Measured (Yes/No), Actionability Assessed (Yes/No), Safety/Risk Discussed (Yes/No), Quantitative Results Reported (Yes/No), Qualitative Feedback Reported (Yes/No), Clinician Preference Reported, Study Limitations Related to XAI, Study Design

### 2.7 Data analysis

#### 2.7.1 Study design classification

This review categorizes each included study into one of four design frameworks based on the Mixed Methods Appraisal Tool (MMAT) (Hong et al., 2018), which also facilitates the risk of bias assessment. **Quantitative descriptive** studies comprise investigations where clinicians evaluate XAI outputs, such as Likert-scale utility ratings for heatmaps, without a formal comparator condition. **Non-randomized quantitative** studies employ structured experimental designs with at least one comparator, typically utilizing a within-subjects paradigm to compare clinician performance with and without XAI support. **Mixed-methods** studies integrate quantitative measures, such as diagnostic accuracy or trust ratings, with qualitative data collection, including think-aloud protocols or semi-structured interviews. Finally, **qualitative** studies rely exclusively on expert evaluations or meetings to explore clinician perceptions. This classification ensures a consistent mapping between the study design and the category-specific quality criteria used for risk of bias assessment.

#### 2.7.2 Risk of bias assessment

This review assesses methodological quality using the MMAT (Hong et al., 2018). While the MMAT is conventionally a quality appraisal instrument, its categorical assessment is utilized in this review to classify studies into three risk-of-bias tiers. Each study is evaluated against five category-specific methodological criteria and rated as “Yes”, “No”, or “Can't tell”. Studies meeting 4–5 criteria are initially classified as Low risk, three criteria as Moderate, and 0–2 criteria as High.

To account for the impact of sample size on generalizability, this review applies an adjusted risk threshold based on the number of clinician evaluators. Although these thresholds are somewhat arbitrary and require borrowing heuristics from adjacent fields, they safeguard against findings driven by individual bias rather than group consensus. Research on content validity (Lynn, 1986) suggest that at least 10 experts are required to stabilize a consensus index, and qualitative interview studies (Guest et al., 2006) indicate that approximately 12 participants are required to reach data saturation and adequately represent group's diversity.

Accordingly, studies involving fewer than *n* < 10 clinicians are classified as High risk regardless of their MMAT score. Studies with 10–19 evaluators are assigned to a Moderate rating, whereas 20 or more evaluators reach an upper threshold where no further size-based adjustments are applied, as this size approximated the statistical power needed for pilot variance estimation. This approach ensures that the review synthesis prioritizes evidence from studies with more representative clinician samples. Details of the risk of bias evaluation for all included studies are provided in Supplementary material S5.

#### 2.7.3 Statistical analysis

Study characteristics were reported as categorical and continuous variables. Categorical variables were reported as numbers and percentages. Continuous variables were reported as mean with standard deviation (SD) if normally distributed and as median with interquartile range (IQR) if not normally distributed. All analyses were performed using R, version 4.3.2 (R Foundation for Statistical Computing).

## 3 Results

### 3.1 Literature search

**Figure 1:**
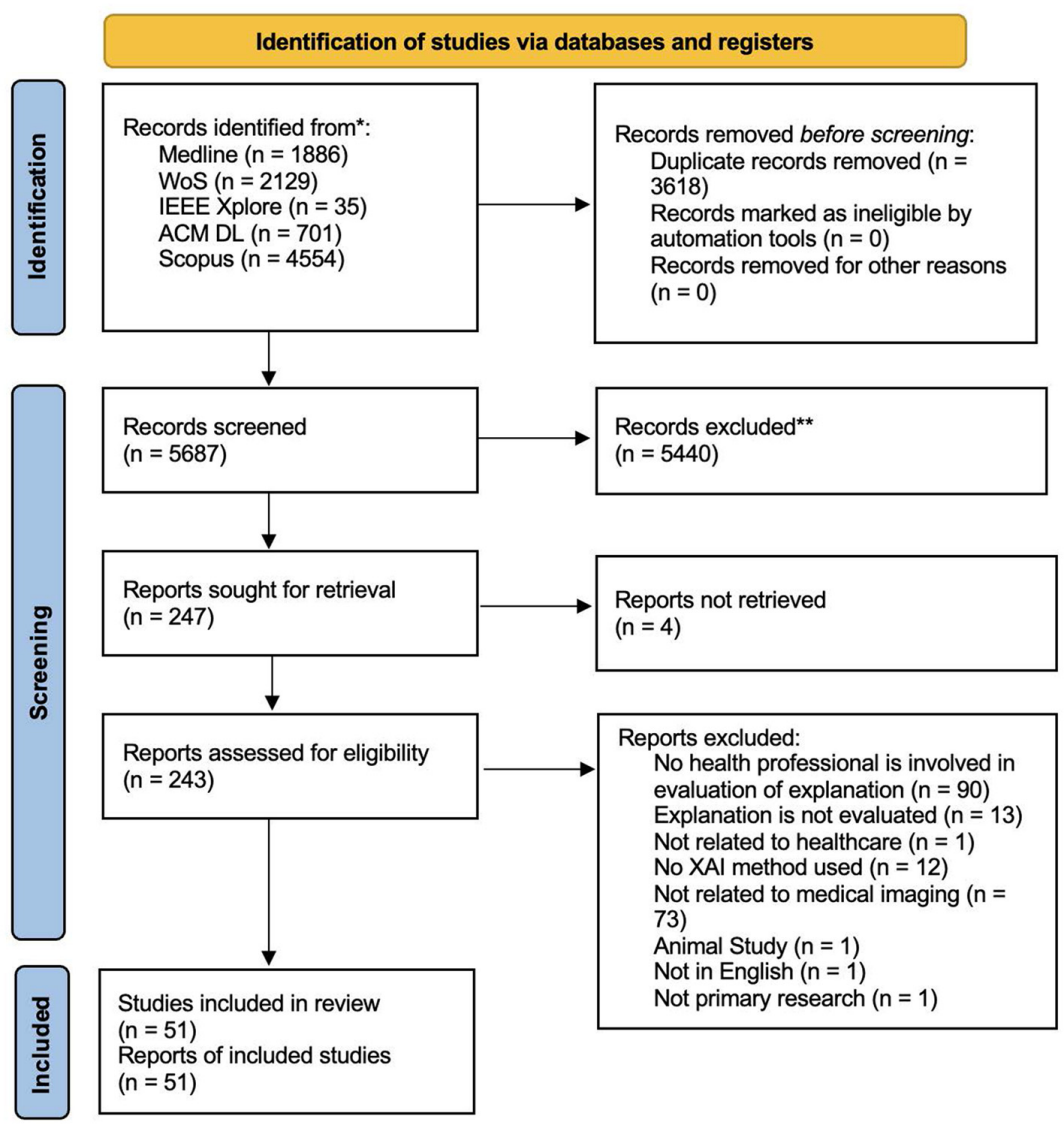
PRISMA flowchart outlining the screening strategy used to identify relevant studies.

A total of 9,305 records were identified across five databases. After removing duplicates, 5,687 references remained for title and abstract screening. Of these, 5,440 articles were deemed irrelevant to the topic and were excluded. A PRISMA flow chart of the data search and study selection process is shown in Figure 1.

Following full-text review, 192 articles were excluded for not meeting the inclusion criteria. The primary reason for exclusion of 90 full-text articles was the lack of involvement of health care professionals in evaluating explainability. A secondary reason for excluding 73 studies was their irrelevance to medical imaging. Based on the predefined criteria, a total of 51 studies were included in the final review in the Supplementary material S3.

Among the 51 included studies, only 12 of the 51 included papers (24%) appeared in journals whose primary audience is practicing clinicians, meaning that the majority of the evidence on how clinicians perceive and use XAI is published in venues that clinicians are unlikely to read routinely (CS/AI venues). This publication pattern may itself contribute to the disconnect between XAI development and clinical adoption.

### 3.2 Chronological analysis of included studies

**Figure 2:**
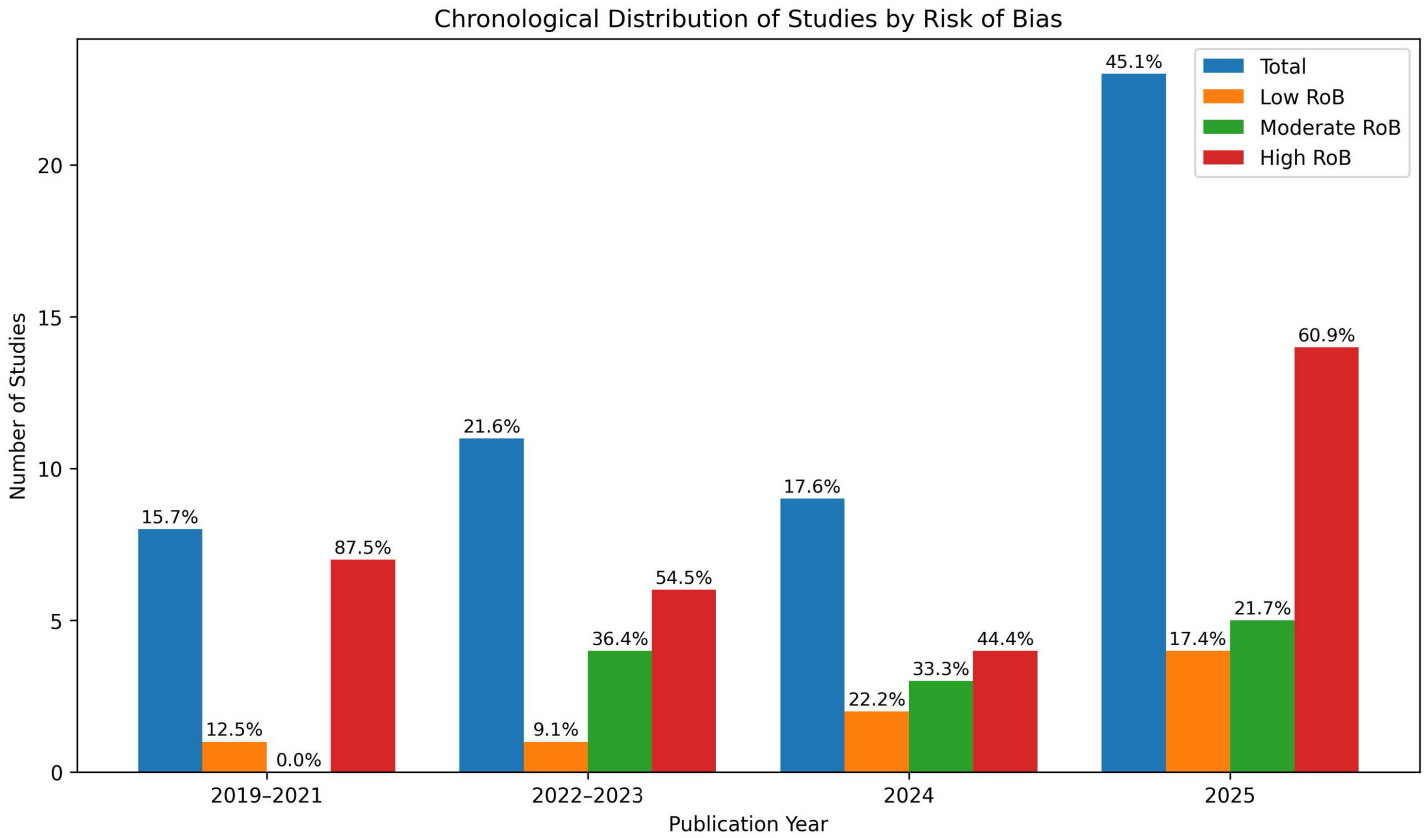
Chronological distribution of included studies by publication year and risk of bias classification. Bars represent the total number of studies published within each time period (2019–2022 combined, 2023, 2024, and 2025), alongside the number of studies categorized as low, moderate, and high risk of bias. Percentages indicate the proportion of studies within each category relative to the total number of included studies, illustrating the temporal trend and methodological quality of publications over time.

A schematic representation of the increase in published articles on clinician-centered evaluation of XAI is shown in Figure 2. The chronological distribution of the included studies demonstrates a clear increase in research activity over time. The earliest studies were published in 2019 (*n* = 2, 3.9%), followed by a modest number in 2020 (*n* = 5, 9.8%). Only one study was identified from 2021 (2.0%), and three studies were published in 2022 (5.9%).

A substantial growth in publications started from 2023 onward, with eight studies (15.7%) in 2023, followed by nine studies (17.6%) in 2024. The majority of the included studies were published in 2025 (*n* = 23, 45.1%). Overall, more than three quarters of the studies (78.4%) published between 2023 and 2025, highlighted a rapid recent expansion of research interest and indicating that clinician-centered evaluation of XAI has become a rapidly developing field.

**Figure 3:**
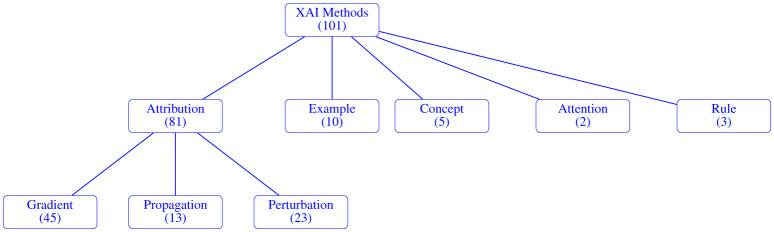
Taxonomy of explainable AI (XAI) methods identified in the 51 included studies (*n* = 101).

Figure 2 depicts a steady rise in the total number of studies from 2019 to 2021 (eight studies) through 2025, where they peak at 45.1% of all included works, highlighting increasing research activity in clinician focused evaluations of XAI in medical imaging. Nonetheless, studies with a low risk of bias remain rare in every year, showing that rigorously conducted, low-bias research has not expanded in step with the overall growth of the field. The share of studies with a moderate risk of bias falls slightly in 2025, pointing to modest gains in study quality. Although clinician-centered evaluation studies are growing rapidly, their quality is still uneven and many are still at high risk of bias. Future work should prioritize increasing the number of low risk of bias studies and strengthening the methodological rigor. Figure 3 presents a taxonomy of explainable AI (XAI) methods to further structure the heterogeneity across studies.

### 3.3 Characteristics of selected studies

Supplementary material S3 summarizes the 51 included studies according to imaging modality, anatomical region, XAI category, clinical specialty, number of clinician evaluators, comparator condition, study design, and risk of bias. The most common imaging modality was X-Ray (*n* = 20), followed by MRI (*n* = 12), optical coherence tomography (OCT; *n* = 5), and CT (*n* = 4), with the remaining studies spanning ultrasound, dermoscopy, endoscopy, histopathology, fundus photography, and facial photography. The largest anatomical groupings include chest imaging and lung tasks combined (13), brain (8), eye (6), breast (4), and prostate (5), of which 4 are MRI studies of prostate lesion detection with radiology readers (Gulum et al., 2024, 2022; Hamm et al., 2023; Trombley et al., 2022).

The XAI category column classifies each study's explanation approach using a high-level taxonomy: *Attribution-based* methods, which assign importance scores to input features or regions, were by far the most prevalent (*n* = 37), followed by *Example-based* (*n* = 11), *Concept-based* (*n* = 6), *Intrinsic* (*n* = 4), *Uncertainty-based* (*n* = 2), and *Rule-based* (*n* = 1); where a study employed multiple categories, all are listed. The number of clinician evaluators ranged from 1 to 118 (median = 10, mean = 16.9), with the majority of studies (*n* = 33, 65%) involving 10 or fewer clinicians and a total of 861 clinicians across all studies. Study designs were predominantly quantitative descriptive (*n* = 20), non-randomized quantitative (*n* = 15), and mixed-methods (*n* = 14), with only three purely qualitative studies. Following sample-size-adjusted MMAT assessment, 31 studies (60%) were rated as high risk of bias, 13 (25%) as moderate, and only 8 (15%) as low, reflecting the generally small evaluator samples characteristic of this field.

### 3.4 XAI methods employed

To provide a more detailed understanding of the specific XAI techniques evaluated in clinical settings, Table 3 organizes the 33 individual XAI methods identified across the included studies into seven categories. The broad *Attribution-based* category showed in Supplementary Table S2 is subdivided into three distinct families based on how attributions are computed: *Gradient-based* methods (e.g., Grad-CAM, Integrated Gradients, SmoothGrad), which derive importance from gradients of the output with respect to the input or intermediate layers; *Propagation-based* methods (e.g., LRP, DeepLIFT, Deep Taylor Decomposition), which backpropagate relevance scores layer by layer through the network; and *Perturbation-based* methods (e.g., SHAP, LIME, Occlusion), which systematically alter inputs and observe the effect on predictions. The remaining categories: *Example-based, Concept-based, Rule-based*, and *Attention-based*, correspond directly to their counterparts in Supplementary Table S2, with the addition of *Attention-based* methods (e.g., transformer attention maps) which were not captured as a separate category at the study level. This finer-grained decomposition reveals that within the dominant attribution-based paradigm, gradient-based approaches account for the largest share (45 of 101 total method usages), suggesting that medical imaging XAI evaluation remains heavily oriented toward visual heatmap-style explanations.

**Table 3:** Taxonomy and frequency of XAI methods employed in the included literature.

Category	XAI Method	Brief Description	Count	Studies
Gradient-based	Grad-CAM	Generates class-discriminative heatmaps using gradients flowing into the final convolutional layer.	20	Al-Bakri et al., 2025; Buijing and Sent, 2025; Dominguez et al., 2023; Ergün et al., 2025; Famiglini et al., 2024; Feng et al., 2025b; Giavina-Bianchi et al., 2023; Gulum et al., 2022, 2024; Hamm et al., 2023; Kinger and Kulkarni, 2025; Liu et al., 2025; Manz et al., 2025; Marmolejo-Saucedo and Kose, 2024; Natali et al., 2023; Pham et al., 2025; Shi et al., 2025; Storås et al., 2025; van den Brandt et al., 2020; Xie et al., 2020
	Integrated Gradients	Attributes importance by accumulating gradients along a path from baseline to input.	6	Gareau et al., 2020; Gulum et al., 2022, 2024; Manz et al., 2025; Singh et al., 2020b; Yang et al., 2025
	Saliency Maps	Visualizes input gradients with respect to the output to highlight influential regions.	5	Chen et al., 2023; Manz et al., 2025; Rezaeian et al., 2025; Singh et al., 2020b; Wysocki et al., 2025
	Grad-CAM++	Extension of Grad-CAM using weighted positive partial derivatives for better localization.	4	Giavina-Bianchi et al., 2023; Kinger and Kulkarni, 2025; Manz et al., 2025; Yang et al., 2025
	SmoothGrad	Reduces noise in saliency maps by averaging over noisy copies of the input.	4	Gulum et al., 2022, 2024; Jin et al., 2024; Singh et al., 2020b
	Guided Backprop.	Combines backpropagation with ReLU masking to visualize specific features.	2	Manz et al., 2025; Singh et al., 2020b
	CAM	Uses global average pooling weights to highlight discriminative regions.	1	Kumar et al., 2021
	Eigen-CAM	Uses principal components of activation maps without relying on gradients.	1	Giavina-Bianchi et al., 2023
	Input x Gradient	Element-wise product of input and gradient; a simple attribution method.	1	Singh et al., 2020b
	Score-CAM	Gradient-free variant using activation-based weights from confidence scores.	1	Giavina-Bianchi et al., 2023
Propagation	DeepLIFT	Compares activations to a reference to assign contribution scores.	4	Gulum et al., 2022, 2024; Singh et al., 2020b; Sümer et al., 2025
	BayLRP	Bayesian LRP combining uncertainty with relevance attribution.	2	Gulum et al., 2024; Trombley et al., 2022
	DeepSHAP	Combines DeepLIFT with Shapley values for deep network attribution.	2	Gulum et al., 2024; Trombley et al., 2022
	LRP	Backpropagates prediction scores to input features via specific rules.	2	Dominguez et al., 2023; Singh et al., 2020b
	CRP	Concept Relevance Propagation; combines LRP with concept-level analysis.	1	Storås et al., 2025
	DeConvNet	Visualizes activations by projecting them back to input space via deconvolution.	1	Singh et al., 2020b
	Deep Taylor	Decomposes output into input relevances using Taylor expansion.	1	Singh et al., 2020b
Perturbation	SHAP	Assigns feature importance based on Shapley values from game theory.	9	Al-Bakri et al., 2025; Bergomi et al., 2025; de Bonis et al., 2025; Feng et al., 2025b; Gulum et al., 2022, 2024; Karagoz et al., 2024; Singh et al., 2020b; Yang et al., 2025
	LIME	Locally interpretable model-agnostic explanations via local surrogates.	6	Dominguez et al., 2023; Ergün et al., 2025; Feng et al., 2025b; Giavina-Bianchi et al., 2023; Karagoz et al., 2024; Kinger and Kulkarni, 2025
	BayLIME	Bayesian extension of LIME incorporating prior knowledge for stability.	2	Gulum et al., 2024; Trombley et al., 2022
	CXPlain	Causal model trained to predict confidence changes from feature masking.	2	Gulum et al., 2024; Trombley et al., 2022
	Occlusion	Systematically masks input regions and measures prediction impact.	2	Singh et al., 2020b; Yang et al., 2025
	Anchors	Produces high-precision if-then rules that locally anchor a prediction.	1	Dominguez et al., 2023
	RISE	Probes the model with randomly masked inputs to generate importance.	1	Highton et al., 2023
Example-based	Counterfactuals	Generates minimal input modifications that would flip the model prediction.	5	Cabitza et al., 2025; Guo et al., 2024; Mertes et al., 2022; Min et al., 2025; Singh et al., 2025
	Influence Functions	Traces predictions back to training examples via leave-one-out approximation.	2	Wysocki et al., 2025; Xie et al., 2020
	ProtoPNet	Classifies by comparing input parts to learned prototypical examples.	2	Gallée et al., 2024; Yazdani et al., 2025
	PCX	Prototype-based Concept Explanations using prototypical concepts.	1	Storås et al., 2025
Concept-based	TCAV	Measures influence of user-defined concepts on model predictions.	3	Hamm et al., 2023; Storås et al., 2025; Xie et al., 2020
	Concept Bottleneck	Intermediate layer predicts human-interpretable concepts before classification.	2	Bashir et al., 2025; Gareau et al., 2020
Attention	Attention Maps	Visualizes transformer or attention weights to show focus regions.	2	Kobayashi et al., 2024; Pham et al., 2025
Rule-based	Rule-based	Generates human-readable if-then rules as decision explanations.	2	Chen et al., 2025; Singh et al., 2025
	AraucanaXAI	Intrinsic framework generating decision-tree-based explanations.	1	Bergomi et al., 2025

### 3.5 XAI impact classification and evaluation

Information related to the user-centered evaluation of XAI methods was extracted from the included studies in six distinct dimensions. These dimensions were derived from and adapted based on an existing XAI evaluation framework to better reflect clinician-focused outcomes in radiology contexts. The first four dimensions, namely: *Understandability/Comprehension, Trust/Confidence, Clinical-Decision-Making/Diagnostic Accuracy*, and *Actionability/Clinical Utility*, are aligned with those defined in the original user-centered evaluation frameworks for XAI (Lopes et al., 2022; Naveed et al., 2024). In addition, a *Clinician Preference* dimension was introduced, as several studies compared multiple explanation methods and explicitly elicited clinicians' preferred approaches. Finally, a *Safety/Automation Bias Mitigation* dimension was included to account for the critical importance of XAI in reducing potential risks and supporting safe decision-making in medical imaging. Table 3 summarizes the six dimensions along with the aspects they assess and the methods used for their evaluation.

These dimensions were integrated into the data extraction form as discussed in Table 4, and in the full text screening data relevant to each one were systematically collected to support structured synthesis and subsequent analysis.

**Table 4:** Summary of core dimensions for clinician-centered evaluation of XAI in medical imaging.

Sr. No	Dimensions	Reference	What is assessed	How assessed
1	Understandability / Comprehension	Lopes et al., 2022; Naveed et al., 2024	whether clinicians can correctly interpret and make sense of the reasoning conveyed by the explanation.	clarity ratings; structured comprehension tasks
2	Trust / Confidence	Lopes et al., 2022; Naveed et al., 2024	evaluates how explanations influence a clinician's willingness to depend on model outputs.	measured through self-reported trust scales; technology acceptance surveys.
3	Clinical-Decision-Making / Diagnostic Accuracy	Lopes et al., 2022; Naveed et al., 2024	whether explanations affect measurable differences in diagnostic performance.	accuracy, sensitivity, or specificity, relative to a baseline condition. (baseline no XAI or no AI)
4	Actionability / Clinical Utility	Lopes et al., 2022; Naveed et al., 2024	Whether an explanation offers enough detail to guide specific clinical decisions.	modifying a treatment strategy or prioritizing a patient's care(with/ without XAI)
5	Clinician Preference		which explanation methods clinicians prefer when several options are presented.	comparative ranking tasks
6	Safety / Automation Bias Mitigation		whether explanations reduce the risk of clinicians uncritically accepting incorrect AI predictions	as compliance rates on deliberately misclassified cases, performance differences between model-correct and model-incorrect conditions

Table 5 maps each included study to six evaluation dimensions, distinguishing between explicit assessment, where a dedicated measurement instrument was used (e.g., Likert scales, diagnostic accuracy metrics, validated trust questionnaires), and implicit assessment, where the dimension was addressed indirectly through qualitative feedback or inferred from other measures. Understandability was the most broadly evaluated dimension, assessed in 41 of 51 studies (80%), with 33 employing explicit instruments such as comprehension Likert scales or structured interpretability tasks. Trust and clinical decision-making were each assessed in 36 studies (71%), and in both cases the vast majority used explicit measures, formal trust scales or technology acceptance models for trust, and before/after diagnostic accuracy comparisons for decision-making. Actionability was assessed in only 23 studies (45%), and almost exclusively through explicit instruments (22 of 23), suggesting that when researchers do evaluate clinical utility they tend to do so rigorously, but that the majority of studies do not consider it at all. Clinician preference was reported in 28 studies (55%), but notably 10 of these (36%) did so only implicitly, for example, by reporting which method yielded the best performance metrics rather than directly soliciting clinician rankings. Only 18 studies explicitly asked clinicians to compare and rank XAI methods, highlighting a gap in understanding which explanation types clinicians actually favor in practice.

**Table 5:** Distribution of Explicit and Implicit Measurements Across Clinical Evaluation Outcomes.

XAI evaluation outcome	Exp.	Explicit studies	Imp.	Implicit studies	Total
Understandability/ comprehension	33	Al-Bakri et al., 2025; Bashir et al., 2025; Bergomi et al., 2025; Buijing and Sent, 2025; Chen et al., 2023, 2025; Dominguez et al., 2023; Feng et al., 2025a,b; Giavina-Bianchi et al., 2023; Gulum et al., 2022, 2024; Hamm et al., 2023; Jin et al., 2024; Karagoz et al., 2024; Kobayashi et al., 2024; Kumar et al., 2021; Lamy et al., 2019; Liu et al., 2025; Manz et al., 2025; Marmolejo-Saucedo and Kose, 2024; Mertes et al., 2022; Niu et al., 2019; Rezaeian et al., 2025; Shi et al., 2025; Singh et al., 2020b; Storås et al., 2025; Sümer et al., 2025; Trombley et al., 2022; van den Brandt et al., 2020; Wysocki et al., 2025; Xie et al., 2020; Yazdani et al., 2025	8	de Bonis et al., 2025; Ergün et al., 2025; Gareau et al., 2020; Highton et al., 2023; Kinger and Kulkarni, 2025; Min et al., 2025; Pham et al., 2025; Yang et al., 2025	41
Trust/confidence	33	Al-Bakri et al., 2025; Buijing and Sent, 2025; Cabitza et al., 2025; Chen et al., 2023; de Bonis et al., 2025; Dominguez et al., 2023; Famiglini et al., 2024; Feng et al., 2025a,b; Giavina-Bianchi et al., 2023; Gulum et al., 2024; Hamm et al., 2023; Highton et al., 2023; Jin et al., 2024; Jungmann et al., 2023; Karagoz et al., 2024; Kinger and Kulkarni, 2025; Kobayashi et al., 2024; Kumar et al., 2021; Liu et al., 2025; Manz et al., 2025; Marmolejo-Saucedo and Kose, 2024; Mertes et al., 2022; Pham et al., 2025; Rezaeian et al., 2025; Shi et al., 2025; Storås et al., 2025; Sümer et al., 2025; Trombley et al., 2022; van den Brandt et al., 2020; Wysocki et al., 2025; Xie et al., 2020; Yazdani et al., 2025	3	Gulum et al., 2022; Guo et al., 2024; Lamy et al., 2019	36
Decision-making/accuracy	34	Cabitza et al., 2024, 2025; Carlile et al., 2020; Chen et al., 2025; Famiglini et al., 2024; Feng et al., 2025a,b; Gallée et al., 2024; Gulum et al., 2022, 2024; Hamm et al., 2023; Jin et al., 2024; Jungmann et al., 2023; Kobayashi et al., 2024; Kumar et al., 2021; Lamy et al., 2019; Liu et al., 2025; Manz et al., 2025; Marmolejo-Saucedo and Kose, 2024; Mertes et al., 2022; Min et al., 2025; Natali et al., 2023; Pham et al., 2025; Rezaeian et al., 2025; Shi et al., 2025; Singh et al., 2020b; Storås et al., 2025; Sümer et al., 2025; Trombley et al., 2022; van den Brandt et al., 2020; Wysocki et al., 2025; Xie et al., 2020; Yang et al., 2025; Yazdani et al., 2025	2	Guo et al., 2024; Singh et al., 2025	36
Actionability/utility	22	Bashir et al., 2025; Bergomi et al., 2025; Cabitza et al., 2025; Chen et al., 2023; Dominguez et al., 2023; Feng et al., 2025a,b; Gallée et al., 2024; Jin et al., 2024; Jungmann et al., 2023; Karagoz et al., 2024; Liu et al., 2025; Marmolejo-Saucedo and Kose, 2024; Mertes et al., 2022; Rezaeian et al., 2025; Shi et al., 2025; Singh et al., 2025; Sümer et al., 2025; van den Brandt et al., 2020; Wysocki et al., 2025; Xie et al., 2020; Yang et al., 2025	1	Cabitza et al., 2024	23
Clinician preference	18	Bergomi et al., 2025; Cabitza et al., 2025; Famiglini et al., 2024; Gallée et al., 2024; Giavina-Bianchi et al., 2023; Gulum et al., 2022, 2024; Hamm et al., 2023; Kinger and Kulkarni, 2025; Kumar et al., 2021; Manz et al., 2025; Mertes et al., 2022; Rezaeian et al., 2025; Singh et al., 2020b, 2025; Storås et al., 2025; Trombley et al., 2022; Wysocki et al., 2025	10	Chen et al., 2023; Feng et al., 2025a; Jin et al., 2024; Karagoz et al., 2024; Liu et al., 2025; Pham et al., 2025; Shi et al., 2025; Sümer et al., 2025; van den Brandt et al., 2020; Xie et al., 2020	28
Safety/automation bias	6	Bergomi et al., 2025; Cabitza et al., 2024, 2025; Gallée et al., 2024; Natali et al., 2023; Rezaeian et al., 2025	5	Buijing and Sent, 2025; Carlile et al., 2020; Guo et al., 2024; Jin et al., 2024; Kobayashi et al., 2024	11

Table 6 summarizes the direction of impact reported by explicitly evaluated studies across all five dimensions. We restrict this analysis to studies that employed dedicated measurement instruments (i.e., explicit assessment), as these provide the most reliable evidence of effect.

**Table 6:** Direction of XAI impact across evaluation dimensions (explicit studies only).

Dimension	N_*exp*_	Positive	Mixed	No impact	Negative
Understandability	33	25 (76%)	6 (18%)	0 (0%)	2 (6%)
Trust	33	24 (73%)	7 (21%)	1 (3%)	1 (3%)
Decision-Making	34	23 (68%)	6 (18%)	4 (12%)	1 (3%)
Actionability	22	20 (91%)	2 (9%)	0 (0%)	0 (0%)
Preference	18	14 (78%)	3 (17%)	0 (0%)	1 (6%)
Safety	6	2 (33%)	1 (17%)	1 (17%)	2 (33%)

#### 3.5.1 Understandability

Among the 33 studies that explicitly measured whether clinicians could comprehend XAI outputs, 25 (76%) reported a positive impact, typically measured through Likert-scale ratings of explanation clarity, structured interpretability tasks, or validated instruments such as the System Usability Scale (Chen et al., 2023). Six studies reported mixed findings, often because understandability varied across XAI methods within the same study. For instance, Wysocki et al. (2025) found that clinicians' actual comprehension varied significantly across six output types and often diverged from self-assessed understanding. Two studies reported negative impact: Gulum et al. (2024) found that radiologists considered attribution maps difficult to interpret for prostate lesion detection, and Rezaeian et al. (2025) demonstrated that progressively increasing explanation complexity significantly reduced understandability.

#### 3.5.2 Trust

Of 33 explicit assessments, 24 (73%) reported that XAI increased clinician trust or confidence, measured through formal trust scales such as Trust in Automation (TiA) Mertes et al. (2022), technology acceptance model (TAM) instruments Chen et al. (2023), or structured plausibility ratings. Seven studies found mixed effects, often conditional on explanation type or clinician expertise, Famiglini et al. (2024) observed that trust effects depended on the granularity of class activation maps, while Buijing and Sent (2025) found trust varied considerably across XAI methods. Notably, Rezaeian et al. (2025) found no significant effect of increasing explanation levels on clinician trust, and Gulum et al. (2024) reported decreased trust due to poor XAI localization quality.

#### 3.5.3 Clinical decision-making

Decision-making impact was explicitly measured in 34 studies, predominantly through pre/post diagnostic accuracy comparisons, sensitivity and specificity analyses, or concordance metrics between clinician and AI predictions. Twenty-three studies (68%) reported positive effects on diagnostic performance, including significant accuracy improvements from 78.8% to 80.9% with example-based explanations (Cabitza et al., 2024) and from 82.5% to 88.5% with AI-assisted neurosurgical diagnosis that included SmoothGrad explanations (Jin et al., 2024). However, this dimension also had the highest proportion of null findings: four studies (12%) found no significant change in diagnostic accuracy with XAI, most notably (Carlile et al., 2020), where emergency physicians did not alter their diagnoses despite receiving AI predictions with Grad-CAM heatmaps during real-time clinical deployment. Six studies reported mixed findings, often revealing that XAI improved accuracy when the model was correct but decreased it when the model predictions where incorrect, an automation bias effect documented by Gallée et al. (2024) and Rezaeian et al. (2025).

#### 3.5.4 Actionability

Actionability was the dimension with the strongest positive consensus: of 22 explicit assessments, 20 (91%) reported that clinicians found XAI outputs clinically useful and potentially integrable into their workflow. Metrics included clinical utility Likert ratings, perceived usefulness scales, self-efficacy measures (Mertes et al., 2022), and structured workflow integration assessments (Bashir et al., 2025). The two mixed findings originated from studies where perceived utility did not uniformly extend across all explanation levels (Jin et al., 2024; Rezaeian et al., 2025). No study reported that XAI had zero or negative actionability, suggesting that when researchers rigorously measure clinical utility, explanations are consistently perceived as valuable. However, the low overall assessment rate (23 of 51 studies, 45%) indicates this dimension is frequently overlooked.

#### 3.5.5 Clinician preference

Among 18 studies that explicitly asked clinicians to compare and rank XAI methods, 14 (78%) identified clear preferences, most commonly through direct ranking tasks or the Explanation Satisfaction Scale (ESS) (Mertes et al., 2022). Counterfactual explanations were preferred over LIME and LRP with large effect sizes in the largest user study (Mertes et al., 2022). SHAP was preferred in 58% of cases in a multi-method comparison (Bergomi et al., 2025). Concept-based approaches were preferred over gradient-based methods for clinical relevance (Storås et al., 2025; Hamm et al., 2023). However, Gulum et al. (2024) found that radiologists rated all XAI methods poorly for prostate MRI, and Rezaeian et al. (2025) observed that the simplest clinical decision support configuration (without detailed explanations) outperformed more complex alternatives, challenging the assumption that more explanation is necessarily better.

#### 3.5.6 Safety

Finally, it was assessed whether studies explicitly considered the safety implications of XAI. Only 11 of 51 studies (22%) addressed this concern at all, with just six studies employing explicit experimental designs (e.g., deliberately including misclassified cases to measure compliance, comparing conditions with escalating explanation levels, or separating accuracy for model-correct vs. model-incorrect predictions). The impact findings for this dimension are different from the predominantly positive results observed for other outcomes: of the six explicitly tested studies, only two reported positive safety effects (Cabitza et al., 2024, 2025), both of which specifically designed mitigation strategies (frictional AI and contrasting “judicial” explanations). Two studies found negative effects, with Bergomi et al. (2025) reporting 86% clinician compliance even on deliberately misclassified cases and Rezaeian et al. (2025) demonstrating that increasing explanation detail decrease diagnostic accuracy. One study found no effect (Natali et al., 2023) and one reported mixed results (Gallée et al., 2024), with explanations boosting confidence in both correct and incorrect predictions. These findings suggest that the overwhelmingly positive impact reported for understandability and trust may not translate to safety, and that standard XAI approaches risk promoting automation bias rather than mitigating it.

### 3.6 Evidence strength

#### 3.6.1 Study designs in lower-risk of bias papers

Eight studies (15.7%) were classified as low risk of bias following sample-size-adjusted MMAT assessment. Their designs span the spectrum from large-scale surveys to controlled experiments and prospective deployments, providing the most reliable evidence base for clinical XAI impact.

Mertes et al. (2022) conducted the most methodologically rigorous evaluation: a between-subjects study with 118 participants randomly assigned to three XAI conditions (GANterfactual(counterfactuals), LIME, LRP), informed by a-priori power analysis and measured through validated instruments (ESS, TiA, DEQ, self-efficacy scales). Counterfactual explanations significantly outperformed alternatives on understanding, trust, and satisfaction, though participants were non-expert, limiting clinical generalisability.

Jin et al. (2024) employed a within-subjects design with 35 neurosurgeons reading 25 brain MRIs under three sequential conditions (no AI, AI prediction, AI + SmoothGrad). AI significantly improved accuracy (82.5% → 87.7%), but the addition of XAI provided no further statistically significant improvement (88.5%), raising questions about the incremental value of saliency-based explanations beyond the AI prediction itself.

Karagoz et al. (2024) surveyed 97 clinicians across multiple specialties, providing the broadest sample representation. High understandability and trust ratings were reported for Grad-CAM explanations, though the survey-only design did not enable causal claims about diagnostic impact.

Rezaeian et al. (2025) involved 28 clinicians and assess the impact of varying levels of AI explanations on their trust and diagnosis accuracy in a breast cancer. This is the only low-risk study reporting predominantly negative effects: increasing explanation complexity reduced understandability, had no effect on trust, and decreased diagnostic agreement, suggesting that explanation design quality matters more than explanation quantity.

Bashir et al. (2025) combined retrospective development with prospective multi-site validation across six hospitals and 46 clinicians, demonstrating high clinical usefulness for concept-based explanations in obstetric ultrasound (segmentation explanations useful in 72.4% of cases).

Carlile et al. (2020) performed the only real-time prospective deployment, embedding AI with Grad-CAM in an emergency department workflow with 45 physicians. Despite high model accuracy, clinicians did not change their diagnostic decisions, providing a critical real-world counterpoint to experimental findings.

Sümer et al. (2025) evaluated a facial phenotyping tool with 31 clinical geneticists, reporting high satisfaction and perceived clinical utility for saliency-based and syndrome-specific explanations.

Wysocki et al. (2025) assessed 27 ophthalmologists' comprehension of six XAI output types, revealing that self-assessed understanding often overestimated actual comprehension, a finding with implications for how understandability should be measured.

#### 3.6.2 AI system maturity and clinical XAI evaluation

To contextualize the strength and translational relevance of the evidence base, all 51 included studies were mapped onto a AI system maturity spectrum reflecting the proximity of each evaluation to real clinical deployment (Table 7). This classification was developed inductively during the review process.

**Table 7:** Technology readiness spectrum of XAI evaluation studies.

Level	Description	N	%	Key characteristic
1	Technical validation	8	16%	Expert visual check by 1–3 specialists; no diagnostic tasks
2	Clinician perception survey	13	25%	Structured questionnaire on XAI outputs; no behavioral measurement
3	Controlled experiment	12	24%	Diagnostic tasks with/without XAI; causal evidence of performance change
4	Mixed-methods evaluation	10	20%	Tasks combined with interviews or think-aloud; captures *how* and *why*
5	Prospective clinical deployment	4	8%	Real or near-real patient encounters in clinical workflow
	Specialist technical	4	8%	Eye-tracking, consensus meetings, iterative feedback

The distribution reveals a substantial translational gap. The largest cluster of studies (Levels 1–2; *n* = 21, 41%) assessed clinician perceptions of XAI without measuring whether explanations actually changed diagnostic behavior. Level 3 studies (*n* = 12, 24%) employed controlled experimental designs, pre-post comparisons, within-subjects conditions, or between-subjects randomization, that provide the strongest causal evidence for XAI impact on clinical decision-making. Level 4 studies (*n* = 10, 20%) enriched experimental evidence with qualitative insights into clinician reasoning processes, offering important context for understanding why certain explanations succeed or fail.

Only four studies (8%) reached Level 5 by evaluating XAI in prospective clinical settings with real patient encounters (Carlile et al., 2020; Liu et al., 2025; Bashir et al., 2025; Pham et al., 2025). Of these, Carlile et al. (2020) found no behavioral change despite high model accuracy, while Liu et al. (2025) reported significant diagnostic improvement across three centers, a divergence that underscores the importance of clinical context, specialty, and explanation design.

Only one study (Mertes et al., 2022) employed a between-subjects randomized design with a-priori power analysis, which represents the gold standard for causal inference in human factors research. The absence of randomized controlled trials in clinical XAI evaluation, despite their ubiquity in clinical medicine, constitutes a significant methodological gap that limits the strength of conclusions about which XAI methods are most clinically impactful.

### 3.7 Clinical impact by explanation type

The central question motivating this review “Which explainable AI methods are most clinically impactful?”, does not yield a single answer. The available evidence indicates that the clinical impact varies according to the type of explanation provided. Each major category is discussed in turn, with an emphasis on findings from studies assessed as having a lower risk of bias.

#### 3.7.1 Attribution-based methods

Gradient-based, propagation-based, and perturbation-based attribution methods, which produce heatmaps, saliency maps, or feature-importance overlays, represented the most common approach in the reviewed literature (37 studies, 72.5%). When evaluated through self-reported measures such as Likert scales and technology acceptance questionnaires, these methods were generally perceived positively: Karagoz et al. (2024) (*N* = 97, low risk) reported high understandability and trust ratings for Grad-CAM explanations across multiple specialties, and Sümer et al. (2025) (*N* = 31, low risk) found similarly favorable perceptions for saliency-based explanations in clinical genetics.

However, the most methodologically rigorous studies paint a different picture. Jin et al. (2024) (*N* = 35 neurosurgeons, low risk) found that SmoothGrad saliency explanations added no incremental diagnostic accuracy beyond the AI prediction itself (82.5% → 87.7% with AI, → 88.5% with AI+XAI), and noted that 31% of decision changes prompted by explanations were incorrect. Rezaeian et al. (2025) (*N* = 28, low risk) demonstrated that progressively increasing explanation detail from AI confidence scores to tumor localization maps to enhanced localization with confidence decreased diagnostic accuracy compared with the simplest AI-only condition and significantly reduced self-reported understandability. Natali et al. (2023) (*N* = 16) observed no improvement in diagnostic accuracy when color-coded saliency maps were added to AI predictions in a crossover reader study of orthopaedists. Carlile et al. (2020) (*N* = 45, low risk), the only prospective real-world deployment in the included literature, found that emergency physicians did not change their diagnostic behavior when presented with Grad-CAM heatmaps during routine COVID-19 chest X-ray interpretation.

Attribution-based methods also performed poorly on automation bias mitigation. Bergomi et al. (2025) (*N* = 10) reported 86% clinician compliance with AI predictions across all explanation formats including SHAP, a rate the authors flagged as indicative of uncritical acceptance. Gallée et al. (2024) (*N* = 6) found that concept-level attribute scores and prototype-based explanations increased clinician confidence not only for correct but also for incorrect predictions. These findings align with evidence from outside the primary synthesis (excluded because the explanations were manually designed rather than AI-generated): Rezazade Mehrizi et al. (2023), in the largest between-subjects experiment identified in our search (92 radiologists, 2,760 decisions), found that neither heatmaps alone nor heatmaps combined with numerical attributes reduced over-reliance on incorrect AI suggestions; and Nagendran et al. (2024) demonstrated through eye-tracking that physicians do not attend more to saliency explanations when AI advice is unsafe.

Not all attribution-based results were negative. Within this category, Famiglini et al. (2024) (*N* = 16) found that lower-level feature CAMs with traditional coloring yielded higher accuracy than higher-level alternatives, suggesting that granularity and visual design matter even within the heatmap paradigm. Likewise, Yang et al. (2025) (*N* = 9) and Jungmann et al. (2023) (*N* = 4) reported diagnostic accuracy improvements in before/after designs, though both had small samples and high risk of bias ratings. The pattern that emerges is that attribution-based explanations can improve perceptions of AI systems but rarely translate into measurable improvements in diagnostic performance or safety, particularly in well-controlled studies.

#### 3.7.2 Example-based methods

In contrast to the mixed attribution-based evidence, example-based methods, counterfactual explanations, similar-case presentations, and contrasting explanation protocols, yielded the most consistently positive results in the included literature (*n* = 11 studies). The strongest evidence comes from Mertes et al. (2022) (*N* = 118, low risk), who conducted a rigorous between-subjects experiment with a prior power analysis comparing GAN-generated counterfactual explanations against LIME and LRP saliency maps. Counterfactuals significantly outperformed both attribution methods on every measured dimension including trust and explanation satisfaction.

Example-based methods also showed unique promise for mitigating automation bias. Cabitza et al. (2025) (*N* = 10) introduced a “judicial” protocol presenting contrasting explanations (evidence both for and against a diagnosis) and found that this improved trust calibration and diagnostic accuracy compared with standard single-prediction formats. Cabitza et al. (2024) (*N* = 16) adopted a “frictional AI” design that presented pro-hoc similar cases rather than post-hoc attribution overlays, achieving a significant positive effect on accuracy, particularly for specialist readers, without inducing complacency. The mechanism underlying these results appears to be that example-based methods engage active comparative reasoning, clinicians must evaluate alternatives rather than passively view highlighted regions which is the deliberative process needed to counteract automation bias.

#### 3.7.3 Concept-based methods

Concept-based explanations, which map model reasoning onto clinically meaningful features such as anatomical landmarks or diagnostic criteria, were evaluated in six studies and showed promising results. Bashir et al. (2025) (*N* = 46, low risk), the largest prospective concept-based evaluation, found that anatomical landmark and image-quality explanations were judged useful in 75% of cases across six hospitals. Storås et al. (2025) (*N* = 5) reported that gastroenterologists explicitly preferred concept-based over gradient-based explanations for clinical relevance.

A particularly informative comparison arises in the domain of prostate MRI, where three included studies evaluated different explanation types with the same clinical specialty (radiology). Gulum et al. (2024) (*N* = 10) found that attribution-based methods were rated poorly across all dimensions, radiologists reported low trust, poor understandability, and no clinical utility, citing inaccurate localization as the primary issue. Its companion study Gulum et al. (2022) (*N* = 10) similarly found no improvement in lesion detection accuracy with post-hoc attribution methods. In contrast, Hamm et al. (2023) (*N* = 7) employed interactive concept-based explanations for the same task and reported positive results for understanding, trust, and PI-RADS scoring accuracy. While these studies differ in design and sample size, the divergent outcomes within the same clinical domain reinforce the conclusion that the type of explanation determines clinical utility at least as much as its mere presence.

#### 3.7.4 Summary

The findings of this review indicate that attribution-based heatmaps and saliency maps are the most frequently studied explanation categories. Across the included studies, these methods are primarily evaluated using clinician perception measures, with limited reporting of outcomes related to decision-making or automation bias mitigation.

Example-based explanation methods are less frequently represented in the literature but are evaluated using both subjective and objective measures. Within the included studies, these methods are assessed in contexts where automation bias mitigation is examined.

Concept-based explanation methods appear in a smaller subset of studies. These approaches map model outputs to clinically meaningful concepts and are evaluated in relation to clinician comprehension and usability.

Across studies focusing on attribution-based methods, self-report measures are commonly used. For example, Wysocki et al. (2025) (*N* = 27, low risk) reports clinician self-assessments of understanding of XAI outputs. Similarly, Nagendran et al. (2024) employs eye-tracking methods to examine the relationship between self-reported usefulness and visual attention to explanations.

## 4 Discussion

### 4.1 Summary of main findings

The findings of this review indicate that while clinician-centered evaluation of XAI in medical imaging is expanding, a significant discrepancy persists between clinicians' subjective perceptions of XAI and its objective impact on diagnostic performance. The available evidence demonstrates that clinical utility is highly dependent on the type of explanation provided.

Attribution-based methods, such as heatmaps and saliency maps, constitute the majority of evaluated approaches and frequently receive high ratings for understandability and trust in survey settings (Karagoz et al., 2024; Sümer et al., 2025). However, more methodologically rigorous evaluations suggest these visual overlays often fail to yield incremental improvements in diagnostic accuracy beyond the AI prediction itself (Jin et al., 2024; Natali et al., 2023). Furthermore, evidence from prospective deployments indicates that such explanations may not influence diagnostic behavior in real-world clinical workflows (Carlile et al., 2020).

Crucially, attribution-based methods appear largely ineffective at mitigating automation bias. Findings suggest that standard attribution approaches may inadvertently increase unwarranted compliance with incorrect AI predictions, particularly as explanation complexity increases (Bergomi et al., 2025; Rezaeian et al., 2025). This indicates that explanations perceived as highly understandable do not inherently safeguard against medical error and may, in some contexts, make erroneous model outputs appear more plausible.

Conversely, example-based and concept-based approaches demonstrate a more robust impact on clinical decision-making. Methods such as counterfactual explanations, similar-case presentations, and contrasting protocols require clinicians to engage in active deliberation rather than passive observation (Mertes et al., 2022; Cabitza et al., 2025, 2024). By aligning more closely with comparative clinical reasoning and specialized medical vocabulary, these approaches show a consistent ability to support diagnostic calibration without inducing the complacency associated with traditional saliency maps (Bashir et al., 2025; Storås et al., 2025). Comparative studies within specific clinical domains further reinforce that concept-based explanations can yield positive effects on understanding and accuracy where attribution-based methods fail (Gulum et al., 2024, 2022; Hamm et al., 2023).

These findings have direct implications for regulatory frameworks that mandate explainability as a prerequisite for clinical AI deployment. The assumption that providing any form of explanation inherently improves safety is empirically unsupported; the underlying mechanism of the explanation is a critical determinant of its clinical utility. Furthermore, because self-reported measures systematically overestimate true comprehension and clinical benefit (Wysocki et al., 2025; Nagendran et al., 2024), perception-based evaluations alone are insufficient for determining actual clinical impact.

### 4.2 Methodological challenges, research gaps, and future directions

Despite progress in applying XAI methods to medical image analysis, a considerable gap persists between the technical generation of explanations and the rigorous evaluation of their clinical utility. Advances in XAI have been argued to be hindered by limited user-centered evaluation, particularly due to shortage of well designed user studies that determine whether explanations genuinely support human decision-making (Pičulin et al., 2025). Consistent with this perspective, our findings indicate that explanation evaluation in medical imaging similarly suffers from insufficient clinician-centered assessment.

The observation that the vast majority of studies demonstrate a moderate or high risk of bias suggests that existing evaluation metrics, frequently adapted from deep learning and computer vision–are often insufficient or lack domain, specific context. These technical metrics may not capture the conceptual and clinical dimensions required for an explanation to be practically useful to a healthcare professional. Furthermore, because explanations are inherently subjective and lack an objective ground truth against which they can be validated, establishing robust, quantitative, and clinically meaningful evaluation criteria remains an open problem. Engaging clinicians early in the study design and metric development phases may help ensure that assessment tools align with actual clinical reasoning and decision-making processes.

The subjective nature of explanations is compounded by the absence of an established framework mapping evaluation methodology to AI system maturity. In the reviewed literature, a substantial proportion of studies relied on perception-based surveys or proof-of-concept evaluations. Conversely, the only real-world prospective study identified in this review found no behavioral change among clinicians (Carlile et al., 2020), a result that might have been anticipated had earlier-stage evaluations incorporated objective performance measures. The field would benefit from explicit guidance outlining when perception-based surveys are sufficient (e.g., early-stage requirements elicitation), when controlled reader studies with diagnostic accuracy endpoints become necessary (e.g., pre-deployment validation), and when prospective real-world trials are required (e.g., regulatory approval). The dominance of self-reported measures is particularly concerning given evidence that they systematically overestimate benefit (Wysocki et al., 2025); moving forward will require a shift toward objective behavioral endpoints, diagnostic accuracy in controlled experiments, decision-time analysis, eye-tracking, and prospective deployment outcomes, that can distinguish genuine clinical impact from perceived utility.

Beyond evaluation design, overall study quality remains a concern, as many investigations are limited by small sample sizes and methodological weaknesses that may compromise the reliability of their findings. The rare application of a priori power analyses (Mertes et al., 2022) and the prevalence of evaluations involving very few clinician evaluators raise the possibility that many reported positive effects are underpowered. Furthermore, the distribution of research effort across explanation types appears misaligned with the evidence of clinical benefit. Example-based and concept-based methods, which have demonstrated the greatest promise for supporting clinical reasoning, remain substantially under-investigated compared to the prevailing focus on attribution-based methods. Finally, head-to-head comparisons of different explanation types within the same clinical context remain rare, with the prostate MRI domain (Gulum et al., 2024, 2022; Hamm et al., 2023) representing a notable exception.

Two critical outcome domains remain almost entirely unaddressed. First, current evaluation endpoints are primarily restricted to clinician cognition or behavior rather than patient benefit, as the measurement of downstream patient outcomes is notably absent. Second, the safety dimension is fundamentally under-investigated. The limited evidence indicating that attribution-based explanations may actually exacerbate rather than mitigate over-reliance on incorrect AI predictions (Bergomi et al., 2025; Rezaeian et al., 2025) requires attention given that regulatory frameworks increasingly mandate explainability as a safety mechanism. Equally absent is any longitudinal evidence: no study examined whether clinicians develop appropriate calibration over time, whether explanation fatigue diminishes engagement, or whether sustained XAI use risks deskilling by discouraging independent reasoning.

Finally, the delayed engagement of healthcare professionals in the development lifecycle likely limits the clinical relevance of XAI systems. Often, clinician involvement is sought only after explanations have already been designed and implemented. Integrating clinicians as collaborative partners throughout all phases of XAI research from conceptualization and study design to evaluation and reporting would facilitate the development of context-sensitive evaluation metrics and contribute to a more meaningful reference standard for explainability. Early engagement provides AI researchers with a clearer understanding of the practical constraints and cognitive requirements of explanations within clinical workflows. Addressing these methodological gaps will ultimately require large-scale, pre-registered, multi-site trials that employ standardized evaluation protocols, objective behavioral endpoints, and sufficient follow-up periods to capture the long-term dynamics of human-AI collaboration in medical image analysis.

### 4.3 Limitations of this study

#### 4.3.1 Scope

This review focuses exclusively on XAI methods applied to medical image analysis. Consequently, it excludes XAI applications for structured clinical data (e.g., electronic health records, genomic profiles), where explanation formats may differ (e.g., tabular feature-importance rankings, natural-language rationales).Similarly, XAI applied to time-series data and clinical text falls outside the present scope. The findings should be interpreted as specific to the imaging context; future reviews synthesizing evidence across diverse data modalities are necessary to provide a comprehensive understanding of clinical XAI impact.

#### 4.3.2 Risk-of-bias assessment

The risk-of-bias assessment evaluated the methodological rigor of clinician-centered evaluations rather than the technical validation of the AI systems. Given the methodological heterogeneity of the included studies, the Mixed Methods Appraisal Tool (MMAT) was selected because it provides design-specific criteria within a single framework. However, MMAT relies on categorical judgments across a limited set of criteria and discourages summary scores. To address these constraints and account for the influence of small sample sizes on generalizability, we implemented non-standard, pragmatic adaptations, specifically, an ordinal risk-of-bias classification and a sample-size adjustment. Although these modifications improved comparability and interpretability, they do not substitute for a fully comprehensive risk of bias framework. There remains scope for future research on more structured and detailed approaches to risk-of-bias assessment. Future reviews may benefit from employing such detailed evaluation tools.

#### 4.3.3 XAI technology readiness classification

The five-level technology readiness taxonomy used to characterize the maturity of evidence in the included studies was developed inductively during the review process rather than adopted from an established framework such as the NASA Technology Readiness Levels or the IDEAL-D model for clinical AI evaluation. While the levels follow a logical progression, from small-panel visual inspection, through perception-only surveys, controlled experiments, mixed-methods evaluation, to prospective clinical deployment, the boundaries between adjacent levels necessarily involve judgment calls. For example, the distinction between a structured survey (Level 2) and a controlled experiment (Level 3) depends on whether clinicians made diagnostic decisions under experimental conditions, a criterion that was not always unambiguously reported in the included articles. Because this classification has not been independently validated, the specific counts per level should be interpreted as indicative of broad patterns in evidence maturity rather than as precise quantitative benchmarks.

#### 4.3.4 Evidence synthesis

A meta-analysis was not feasible due to the substantial heterogeneity of the study with respect to imaging modalities, clinical specialties, explanation types, experimental designs, and outcome measures. The resulting narrative synthesis inherently involves interpretive judgment. The classification of evaluation dimensions and the assignment of impact direction were performed by the review team based on reported methods; other reviewers might reach different classifications for borderline cases.

As the included studies employ heterogeneous evaluation designs, outcome measures, and reporting standards, the comparison of explanation methods across studies should be interpreted with caution. No formal cross-study normalization or statistical comparison was feasible, limiting the ability to draw definitive conclusions regarding the relative performance of different explanation approaches.

Among the six evaluation dimensions, the first four are aligned with those defined in the original user-centered evaluation frameworks for XAI (Lopes et al., 2022; Naveed et al., 2024), while the last two were identified iteratively during the review process rather than drawn from a pre-existing validated taxonomy. The *Clinician Preference* dimension was introduced, as several studies compared multiple explanation methods and explicitly elicited clinicians' preferred approaches. Similarly *Safety/Automation Bias Mitigation* dimension was included to account for the critical importance of XAI in reducing potential risks and supporting safe decision-making in medical imaging.As two of these six evaluation dimensions were not drawn from a pre-existing validation frameworks, potentially limiting comparability with future reviews.

A further methodological consideration is that, given the volume of retrieved records, AI-assisted tools were used to support a large portion of the data extraction process for papers that proceeded to full-text screening. Although all included studies were subsequently verified and evaluated manually (SUD, MI), this workflow introduces the possibility of confirmation bias. While we mitigated this risk through independent cross-checking of key judgements, we cannot exclude that the AI-assisted extraction influenced the final interpretations.

#### 4.3.5 Reviewer expertise

This review is subject to the same limitation it identifies in the included literature: the review team did not include extensive clinical representation across all specialties covered by the included studies, and domain experts were involved at later stages of the review process rather than from its inception. As a consequence, the classification of evaluation dimensions, the interpretation of clinical relevance, and the assessment of whether specific XAI outputs align with established diagnostic reasoning may reflect a predominantly technical rather than clinical perspective. While we sought clinical input to validate key judgements, an earlier and broader clinical involvement in the design of the data extraction framework and the formulation of evaluation categories might have yielded a more clinically grounded synthesis. This limitation reinforces the recommendation that future XAI research should integrate clinicians as equal partners from the earliest stages of study design.

## 5 Conclusions

This systematic review indicates that while clinician-centered assessments of explainable AI (XAI) in medical imaging are expanding, robust evidence demonstrating measurable clinical benefit remains limited. Current evaluation practices are frequently constrained by insufficient clinical involvement. Integrating healthcare professionals in all phases of XAI research, from conceptualization and study design to evaluation and reporting, is essential to establish a clinically meaningful framework to assess explainability.

Among the 51 included studies, attribution-based heatmaps were the most frequently examined explanation modality. Evidence from methodologically rigorous evaluations suggests that, while these methods are associated with increased perceived understanding and trust, their impact on diagnostic accuracy and automation bias mitigation remains limited or inconclusive. In comparison, example-based explanations (e.g., counterfactual or contrastive approaches) and concept-based explanations show potential for clinical utility, as they appear to align more closely with comparative clinical reasoning processes. Example-based and concept-based methods show positive signals across the included studies; however, direct comparison across methods is limited by the lack of standardized evaluation frameworks and controlled cross-study normalization.

To bridge the translational gap between technical development and clinical adoption, future research must prioritize pre-registered, adequately powered, multi-site evaluations. These studies should: (i) measure objective behavioral endpoints, such as diagnostic accuracy and error detection; (ii) explicitly test safety and automation-bias mechanisms under deliberately incorrect model outputs; and (iii) involve clinicians early in the explanation design process to ensure clinical relevance, usability, and seamless workflow integration.

## Data Availability

The original contributions presented in the study are included in the article/Supplementary material, further inquiries can be directed to the corresponding author.
