# Supplementary material for: Which explainable AI methods in medical imaging are clinically impactful? A systematic literature review addressing the clinician's perspective
Source: Front Artif Intell. 2026 May 29;9:1819422. doi: 10.3389/frai.2026.1819422 (PMC13260647; doi:10.3389/frai.2026.1819422)
Supplement: Supplementary file 1 [file Supplementary_file_1.pdf]

# Supplementary Material

## 1 RELATED SURVEYS

Table S1: Comparative overview of related surveys on XAI in medical imaging

| Ref. | Systematic review or not? | Used specific disease or anatomical region? | Addressing XAI method, technique or category? | Addressed clinician's evaluation? | Distinctive contribution |
|---|---|---|---|---|---|
| (1) | yes | no | 18 explainability method | no | Identified 18 XAI methods; foundations and limitations. |
| (2) | yes | no | 4 categories | no | Comprehensive review of XAI methods and clinical implications. |
| (3) | no | no | 3 categories | no | Classified 223 studies; compared pros and cons. |
| (4) | no | no | 5 categories | no | Mini-review of XAI applications and multi modal fusion. |
| (5) | no | no | 7 categories | no | Reviewed approaches, deployment challenges, and research gaps. |
| (6) | no | no | 9 different techniques discussed | no | Categorized XAI algorithms; analyzed performance and challenges. |
| (7) | no | no | 9 different methods identifies | no | Summarized interpretability methods and evaluation strategies. |
| (8) | no | no | 4 classes of techniques discussed | no | Taxonomy of XAI in biomedical imaging; future directions. |
| (9) | no | no | 12 methods | no | Categorized interpretable ML methods in healthcare. |
| (10) | no | no | 4 categories | no | Classified non-visual XAI methods; strengths and targets. |
| (11) | no | cancer | 7 methods | no | Reviewed MRI cancer XAI; clinician expectation gap. |
| (12) | no | COVID-19 | 3 categories | no | Discussed XAI evaluation frameworks and best practices. |
| (13) | yes | dementia | 18 methods | no | Analyzed 92 studies: modalities, models, methods. |
| (14) | no | no | 4 categories | no | Surveyed XAI types, datasets, and evaluation metrics. |
| (15) | no | no | 13 methods | no | Identified interpretability limitations and research gaps. Discuss few examples of quantitative and qualitative explanation of explainability. |
| (16) | yes | nuclear medicine | 10 models | no | Highlighted lack of consensus on clinical XAI deployment. |
| (17) | no | cardiac images | 20 models | no | Proposed taxonomy; early-stage cardiac imaging XAI. |
| (18) | yes | no | 18 techniques | no | Quantitative overview of DL explainability in radiology. |
| (19) | no | no | n/a | no | Mapped XAI techniques across diagnostic imaging in scoping review. |
| (20) | no | no | c5 techniques | no | Reviewed XAI effectiveness in clinical relevance. |

*Continued on next page*

| Ref. | Systematic review or not? | Used specific disease or anatomical region? | Addressing XAI method, technique or category? | Addressed clinician's evaluation? | Distinctive contribution |
|---|---|---|---|---|---|
| (21) | yes | no | n/a | no | Analyzed LIME applications in healthcare. |
| (22) | no | no | n/a | no | Identified a number of obstacles associated with XAI in the healthcare area, including System Evaluation, Organizational, Legal, socio relational, and Communication issues. |
| (23) | n | no | n/a | no | Provides a review for the use of XAI in context of Internet of Health Things. |

## REFERENCES


[1]Muhammad D, Bendechache M. Unveiling the black box: A systematic review of Explainable Artificial Intelligence in medical image analysis. Computational and structural biotechnology journal. 2024;24:542-60.

[2]Ahmed F, Naz NS, Khan S, Rehman AU, Ismael WM, Khan MA. Explainable artificial intelligence (XAI) in medical imaging: a systematic review of techniques, applications, and challenges. BMC Medical Imaging. 2026.

[3]Van der Velden BH, Kuijf HJ, Gilhuijs KG, Viergever MA. Explainable artificial intelligence (XAI) in deep learning-based medical image analysis. Medical image analysis. 2022;79:102470.

[4]Yang G, Ye Q, Xia J. Unbox the black-box for the medical explainable AI via multi-modal and multi-centre data fusion: A mini-review, two showcases and beyond. Information Fusion. 2022;77:29-52.

[5]Singh A, Sengupta S, Lakshminarayanan V. Explainable deep learning models in medical image analysis. Journal of imaging. 2020;6(6):52.

[6]Chaddad A, Peng J, Xu J, Bouridane A. Survey of explainable AI techniques in healthcare. Sensors. 2023;23(2):634.

[7]Salahuddin Z, Woodruff HC, Chatterjee A, Lambin P. Transparency of deep neural networks for medical image analysis: A review of interpretability methods. Computers in biology and medicine. 2022;140:105111.

[8]Nazir S, Dickson DM, Akram MU. Survey of explainable artificial intelligence techniques for biomedical imaging with deep neural networks. Computers in Biology and Medicine. 2023;156:106668.

[9]Band SS, Yarahmadi A, Hsu CC, Biyari M, Sookhak M, Ameri R, et al. Application of explainable artificial intelligence in medical health: A systematic review of interpretability methods. Informatics in Medicine Unlocked. 2023;40:101286.

[10]Borys K, Schmitt YA, Nauta M, Seifert C, Krämer N, Friedrich CM, et al. Explainable AI in medical imaging: An overview for clinical practitioners–Beyond saliency-based XAI approaches. European journal of radiology. 2023;162:110786.

[11]Gulum MA, Trombley CM, Kantardzic M. A review of explainable deep learning cancer detection models in medical imaging. Applied Sciences. 2021;11(10):4573.

[12]Fuhrman JD, Gorre N, Hu Q, Li H, El Naqa I, Giger ML. A review of explainable and interpretable AI with applications in COVID-19 imaging. Medical Physics. 2022;49(1):1-14.

[13]Martin SA, Townend FJ, Barkhof F, Cole JH. Interpretable machine learning for dementia: a systematic review. Alzheimer's & Dementia. 2023;19(5):2135-49.

[14]Patrício C, Neves JC, Teixeira LF. Explainable deep learning methods in medical image classification: A survey. ACM Computing Surveys. 2023;56(4):1-41.
[15]Ennab M, Mcheick H. Enhancing interpretability and accuracy of AI models in healthcare: a comprehensive review on challenges and future directions. Frontiers in Robotics and AI. 2024;11:1444763.
[16]De Vries BM, Zwezerijnen GJ, Burchell GL, van Velden FH, Menke-van der Houven van Oordt CW, Boellaard R. Explainable artificial intelligence (XAI) in radiology and nuclear medicine: a literature review. Frontiers in medicine. 2023;10:1180773.
[17]Salih A, Boscolo Galazzo I, Gkontra P, Lee A, Lekadir K, Raisi-Estabragh Z, et al.. Explainable artificial intelligence and cardiac imaging: toward more interpretable models. Circ Cardiovasc Imaging. Lippincott Williams & Wilkins Hagerstown, MD; 2023.
[18]Groen AM, Kraan R, Amirkhan SF, Daams JG, Maas M. A systematic review on the use of explainability in deep learning systems for computer aided diagnosis in radiology: Limited use of explainable AI? European journal of radiology. 2022;157:110592.
[19]Champendal M, Müller H, Prior JO, Dos Reis CS. A scoping review of interpretability and explainability concerning artificial intelligence methods in medical imaging. European journal of radiology. 2023;169:111159.
[20]Bhati D, Neha F, Amiruzzaman M. A survey on explainable artificial intelligence (XAI) techniques for visualizing deep learning models in medical imaging. Journal of Imaging. 2024;10(10):239.
[21]Hassan SU, Abdulkadir SJ, Zahid MSM, Al-Selwi SM. Local interpretable model-agnostic explanation approach for medical imaging analysis: A systematic literature review. Computers in Biology and Medicine. 2025;185:109569.
[22]Bharati S, Mondal MRH, Podder P. A review on explainable artificial intelligence for healthcare: Why, how, and when? IEEE Transactions on Artificial Intelligence. 2023;5(4):1429-42.
[23]Bharati S, Mondal MRH, Podder P, Kose U. Explainable artificial intelligence (XAI) with IoHT for smart healthcare: A review. Interpretable Cognitive Internet of Things for Healthcare. 2023:1-24.
