# Supplementary material for: Which explainable AI methods in medical imaging are clinically impactful? A systematic literature review addressing the clinician's perspective
Source: Front Artif Intell. 2026 May 29;9:1819422. doi: 10.3389/frai.2026.1819422 (PMC13260647; doi:10.3389/frai.2026.1819422)
Supplement: Supplementary file 2 [file Supplementary_file_2.pdf]

Supplementary Material

1 SEARCH TERMS

Table S1: Full Electronic Database Search Strategies and Results (November 17, 2025)

Database	Search Strategy	Hits
**Ovid MEDLINE(R)**	#1 exp ”Physicians”/ or (”radiologist*” or (”medical*” adj3 (”expert*” or ”specialist*”)) or ”oncologist*” or ”doctor*” or ”surgeon*” or ”clinician*” or ”physician*” or ”anesthesiologist*” or ”anaesthesiologist*” or ”cardiologist*” or ”gastroenterologist*” or ”enterologist*” or ”general practitioner*” or ”geriatrician*” or ”gynecologist*” or ”gynaecologist*” or ”hospitalist*” or ”nephrologist*” or ”neurologist*” or ”obstetrician*” or ”obstaetrician*” or ”ophthalmologist*” or ”otolaryngologist*” or ”pathologist*” or ”pediatrician*” or ”paediatrician*” or ”neonatologist*” or ”obstetrician*” or ”pulmonologist*” or ”rheumatologist*” or ”reumatologist*” or ”neurosurgeon*” or ”urologist*” or ”epidemiologist*” or ”midwife*” or ”midwive*” or ”resident*” or ”residenc*” or ”consultant*” or ”intern” or ”interns” or ”internship*” or ”allergist*” or ”subspecialist*” or ”immunologist*” or ”anesthesist*” or ”anesthesiologist*” or ”anaesthesist*” or ”anaesthesiologist*” or ”specialist*” or ”cardiologist*” or ”dermatologist*” or ”toxicologist*” or ”general practitioner*” or ”geriatrist*” or ”gerontologist*” or ”nephrologist*” or ”internist*” or ”otolaryngologist*” or ”laryngologist*” or ”otologist*” or ”neurotologist *” or ”pathologist*” or ”psychiatrist*” or ”neurologist*” or ”neuroradiologist*”).ti,ab,kf.<br>#2 (”explaina*” or ”xai” or ”interpreta*”).ti,ab,kf.<br>#3 exp ”Artificial Intelligence”/ or (”artificial intelligen*” or ”ai” or ”machine learning” or ”deep learning” or ”chatgpt” or ”chat gpt” or ”llm” or ”llms” or ”large language model*” or ”transformer*”).ti,ab,kf.<br>#4 ”Surveys and Questionnaires”/ or exp ”Self Report”/ or ”Interview”.pt. or exp ”Interviews as Topic”/ or (”survey*” or ”evaluati*” or ”quantitative analys*” or ”qualitative analys*” or ”interview*” or ”self report*” or ”questionnaire*” or (”user*” adj3 (”stud*” or ”perspective*” or ”preferen*”))).ti,ab,kf.<br>#5 1 and 2 and 3 and 4	1,886

Table S1 – continued from previous page

Database	Search Strategy	Hits
**Web of Science**	#1 TS=(”radiologist*” or (”medical*” NEAR/3 ”expert*”) or ”oncologist*” or ”doctor*” or ”surgeon*” or ”clinician*” or ”physician*” or ”anesthesiologist*” or ”anaesthesiologist*” or ”cardiologist*” or ”gastroenterologist*” or ”enterologist*” or ”general practitioner*” or ”geriatrician*” or ”gynecologist*” or ”gynaecologist*” or ”hospitalist*” or ”nephrologist*” or ”neurologist*” or ”obstetrician*” or ”obstaetrician*” or ”ophthalmologist*” or ”otolaryngologist*” or ”pathologist*” or ”pediatrician*” or ”paediatrician*” or ”neonatologist*” or ”obstetrician*” or ”pulmonologist*” or ”rheumatologist*” or ”reumatologist*” or ”neurosurgeon*” or ”urologist*” or ”epidemiologist*” or ”midwife*” or ”midwive*” or ”resident*” or ”residenc*” or ”consultant*” or ”intern” or ”interns” or ”internship*” or ”allergist*” or ”subspecialist*” or ”immunologist*” or ”anesthesist*” or ”anesthesiologist*” or ”anaesthesist*” or ”anaesthesiologist*” or ”specialist*” or ”cardiologist*” or ”dermatologist*” or ”toxicologist*” or ”general practitioner*” or ”geriatrist*” or ”gerontologist*” or ”nephrologist*” or ”internist*” or ”otolaryngologist*” or ”laryngologist*” or ”otologist*” or ”neurotologist *” or ”pathologist*” or ”psychiatrist*” or ”neurologist*” or ”neuroradiologist*”)<br>#2 TS=(”explaina*” or ”xai” or ”interpreta*”)<br>#3 TS=(”artificial intelligen*” or ”ai” or ”machine learning” or ”deep learning” or ”chatgpt” or ”chat gpt” or ”llm” or ”llms” or ”large language model*” or ”transformer*”)<br>#4 TS=(”survey*” or ”evaluati*” or ”quantitative analys*” or ”qualitative analys*” or ”interview*” or ”self report*” or ”questionnaire*” or (”user*” NEAR/3 (”stud*” or ”perspective*” or ”preferen*”)))<br>#5 #4 AND #3 AND #2 AND #1	2,129
**Scopus**	#1 TITLE-ABS-KEY (”explaina*” OR ”xai” OR ”interpreta*”)<br>#2 TITLE-ABS-KEY (”artificial intelligen*” OR ”ai” OR ”machine learning” OR ”deep learning” OR ”chatgpt*” OR ”chat gpt” OR ”llm” OR ”llms” OR ”large language model*” OR ”transformer*”)<br>#3 TITLE-ABS-KEY (”survey*” OR ”evaluati*” OR ”quantitative analys*” OR ”qualitative analys*” OR ”interview*” OR ”self report*” OR ”questionnaire*” OR ”user stud*” OR ”perspective*” OR ”preferen*”)<br>#4 TITLE-ABS-KEY (”radiologist*” OR ”medical expert*” OR ”oncologist*” OR ”doctor*” OR ”surgeon*” OR ”clinician*” OR ”physician*” OR ”anesthesiologist*” OR ”anaesthesiologist*” OR ”cardiologist*” OR ”gastroenterologist*” OR ”enterologist*” OR ”general practitioner*” OR ”geriatrician*” OR ”gynecologist*” OR ”gynaecologist*” OR ”hospitalist*” OR ”nephrologist*” OR ”neurologist*” OR ”obstetrician*” OR ”obstaetrician*” OR ”ophthalmologist*” OR ”otolaryngologist*” OR ”pathologist*” OR ”pediatrician*” OR ”paediatrician*” OR ”neonatologist*” OR ”obstetrician*” OR ”pulmonologist*” OR ”rheumatologist*” OR ”reumatologist*” OR ”neurosurgeon*” OR ”urologist*” OR ”epidemiologist*” OR ”midwife*” OR ”midwive*” OR ”resident*” OR ”residenc*” OR ”consultant*” OR ”intern” OR ”interns” OR ”internship*” OR ”allergist*” OR ”subspecialist*” OR ”immunologist*” OR ”anesthesist*” OR ”anesthesiologist*” OR ”anaesthesist*” OR ”anaesthesiologist*” OR ”specialist*” OR ”cardiologist*” OR ”dermatologist*” OR ”toxicologist*” OR ”general practitioner*” OR ”geriatrist*” OR ”gerontologist*” OR ”nephrologist*” OR ”internist*” OR ”otolaryngologist*” OR ”laryngologist*” OR ”otologist*” OR ”neurotologist*” OR ”pathologist*” OR ”psychiatrist*” OR ”neurologist*” OR ”neuroradiologist*”)<br>#5 #1 AND #2 AND #3 AND #4	4,554

Table S1 – continued from previous page

Database	Search Strategy	Hits
**ACM Digital Library**	[[All: ”radiologist*”] OR [All: ”medical expert*”] OR [All: ”oncologist*”] OR [All: ”doctor*”] OR [All: ”surgeon*”] OR [All: ”clinician*”] OR [All: ”physician*”] OR [All: ”anesthesiologist*”] OR [All: ”anaesthesiologist*”] OR [All: ”cardiologist*”] OR [All: ”gastroenterologist*”] OR [All: ”enterologist*”] OR [All: ”general practitioner*”] OR [All: ”geriatrician*”] OR [All: ”gynecologist*”] OR [All: ”gynaecologist*”] OR [All: ”hospitalist*”] OR [All: ”nephrologist*”] OR [All: ”neurologist*”] OR [All: ”obstetrician*”] OR [All: ”obstaetrician*”] OR [All: ”ophthalmologist*”] OR [All: ”otolaryngologist*”] OR [All: ”pathologist*”] OR [All: ”pediatrician*”] OR [All: ”paediatrician*”] OR [All: ”neonatologist*”] OR [All: ”obstetrician*”] OR [All: ”pulmonologist*”] OR [All: ”rheumatologist*”] OR [All: ”reumatologist*”] OR [All: ”neurosurgeon*”] OR [All: ”urologist*”] OR [All: ”epidemiologist*”] OR [All: ”midwife*”] OR [All: ”midwive*”] OR [All: ”resident*”] OR [All: ”residenc*”] OR [All: ”consultant*”] OR [All: ”intern*”] OR [All: ”interns*”] OR [All: ”internship*”] OR [All: ”allergist*”] OR [All: ”subspecialist*”] OR [All: ”immunologist*”] OR [All: ”anesthesist*”] OR [All: ”anesthesiologist*”] OR [All: ”anaesthesist*”] OR [All: ”anaesthesiologist*”] OR [All: ”specialist*”] OR [All: ”cardiologist*”] OR [All: ”dermatologist*”] OR [All: ”toxicologist*”] OR [All: ”general practitioner*”] OR [All: ”geriatrist*”] OR [All: ”gerontologist*”] OR [All: ”nephrologist*”] OR [All: ”internist*”] OR [All: ”otolaryngologist*”] OR [All: ”laryngologist*”] OR [All: ”otologist*”] OR [All: ”neurotologist *”] OR [All: ”pathologist*”] OR [All: ”psychiatrist*”] OR [All: ”neurologist*”] OR [All: ”neuroradiologist*”]] AND [[All: ”explaina*”] OR [All: ”xai”] OR [All: ”interpreta*”]] AND [[All: ”artificial intelligen*”] OR [All: ”ai”] OR [All: ”machine learning”] OR [All: ”deep learning”] OR [All: ”chatgpt*”] OR [All: ”chat gpt”] OR [All: ”llm”] OR [All: ”llms”] OR [All: ”large language model*”] OR [All: ”transformer*”]] AND [[All: ”survey*”] OR [All: ”evaluati*”] OR [All: ”quantitative analys*”] OR [All: ”qualitative analys*”] OR [All: ”interview*”] OR [All: ”self report*”] OR [All: ”questionnaire*”] OR [All: ”user stud*”] OR [All: ”perspective*”] OR [All: ”preferen*”]]	701

Table S1 – continued from previous page

Database	Search Strategy	Hits
**IEEE Xplore**	(”All Metadata”:radiologist OR ”All Metadata”:”medical expert*” OR ”All Metadata”:oncologist OR ”All Metadata”:doctor OR ”All Metadata”:surgeon OR ”All Metadata”:clinician OR ”All Metadata”:physician OR ”All Metadata”:anesthesiologist OR ”All Metadata”:anaesthesiologist OR ”All Metadata”:cardiologist OR ”All Metadata”:gastroenterologist OR ”All Metadata”:enterologist OR ”All Metadata”:”general practitioner*” OR ”All Metadata”:geriatrician OR ”All Metadata”:gynecologist OR ”All Metadata”:gynaecologist OR ”All Metadata”:hospitalist OR ”All Metadata”:nephrologist OR ”All Metadata”:neurologist OR ”All Metadata”:obstetrician OR ”All Metadata”:obstaetrician OR ”All Metadata”:ophthalmologist OR ”All Metadata”:otolaryngologist OR ”All Metadata”:pathologist OR ”All Metadata”:pediatrician OR ”All Metadata”:paediatrician OR ”All Metadata”:neonatologist OR ”All Metadata”:obstetrician OR ”All Metadata”:pulmonologist OR ”All Metadata”:rheumatologist OR ”All Metadata”:reumatologist OR ”All Metadata”:neurosurgeon OR ”All Metadata”:urologist OR ”All Metadata”:epidemiologist OR ”All Metadata”:midwife OR ”All Metadata”:midwive OR ”All Metadata”:resident OR ”All Metadata”:residenc OR ”All Metadata”:consultant OR ”All Metadata”:intern OR ”All Metadata”:interns OR ”All Metadata”:internship OR ”All Metadata”:allergist OR ”All Metadata”:subspecialist OR ”All Metadata”:immunologist OR ”All Metadata”:anesthesist OR ”All Metadata”:anesthesiologist OR ”All Metadata”:anaesthesist OR ”All Metadata”:anaesthesiologist OR ”All Metadata”:specialist OR ”All Metadata”:cardiologist OR ”All Metadata”:dermatologist OR ”All Metadata”:toxicologist OR ”All Metadata”:”general practitioner*” OR ”All Metadata”:geriatrist OR ”All Metadata”:gerontologist OR ”All Metadata”:nephrologist OR ”All Metadata”:internist OR ”All Metadata”:otolaryngologist OR ”All Metadata”:laryngologist OR ”All Metadata”:otologist OR ”All Metadata”:neurotologist OR ”All Metadata”:pathologist OR ”All Metadata”:psychiatrist OR ”All Metadata”:neurologist OR ”All Metadata”:neuroradiologist) AND (”All Metadata”:explaina OR ”All Metadata”:xai OR ”All Metadata”:interpreta) AND (”All Metadata”:”artificial intelligen*” OR ”All Metadata”:ai OR ”All Metadata”:”machine learning” OR ”All Metadata”:”deep learning” OR ”All Metadata”:chatgpt OR ”All Metadata”:”chat gpt” OR ”All Metadata”:llm OR ”All Metadata”:llms OR ”All Metadata”:”large language model*” OR ”All Metadata”:transformer) AND (”All Metadata”:survey OR ”All Metadata”:evaluati OR ”All Metadata”:”quantitative analys*” OR ”All Metadata”:”qualitative analys*” OR ”All Metadata”:interview OR ”All Metadata”:”self report*” OR ”All Metadata”:questionnaire OR ”All Metadata”:”user stud*” OR ”All Metadata”:perspective OR ”All Metadata”:preferen)	35
