# Supplementary material for: Which explainable AI methods in medical imaging are clinically impactful? A systematic literature review addressing the clinician's perspective
Source: Front Artif Intell. 2026 May 29;9:1819422. doi: 10.3389/frai.2026.1819422 (PMC13260647; doi:10.3389/frai.2026.1819422)
Supplement: Supplementary file 3 [file Supplementary_file_3.pdf]

Supplementary Material

1 FULL TEXT SCREENING

Table S1. Claude vs human reviewers (AL and SUD): Full Text Screening.

Comparison	N	Exact agreement	Cohen's κ
Claude vs AL	64	82.8%	0.60
Claude vs SUD	59	89.8%	0.71

On full screened papers by humans and Claude, Claude endorsed inclusion more often than either human:

- **vs SUD:** SUD 10/59 included (16.9%), Claude 16/59 (27.1%)—6 more inclusions by Claude.
- **vs AL:** AL 18/64 included (28.1%), Claude 21/64 (32.8%)—3 more inclusions by Claude.

Disagreements were therefore asymmetric, with several records excluded by the human but Included by Claude. All papers included by Claude were subsequently reviewed by SUD, and data extraction was corrected where necessary. Full texts that were not initially reviewed by a human reviewer but were excluded by Claude were double-checked by M.I. to verify the reason for exclusion.

2 INCLUDED STUDIES

Table S2: Systematic Summary of Included Studies and Experimental Characteristics

Ref.	Mod.	Region	XAI Category	Specialty	N	Comparator	Study Design	Risk of Bias
(1)	XR	Chest	Attribution-based; Intrinsic	Infectious Diseases; Emergency Medicine	10	Other XAI methods	Mixed-meth. (Likert quest. + think-aloud)	Moderate
(2)	XR	Spine	Example-based	Orthopaedics	16	Without XAI	Non-rand. quant. (before/after)	Moderate
(3)	XR	Chest	Example-based	Radiology	2	No comparator	Quant. desc. (expert Likert)	High
(4)	MRI	Brain	Attribution-based	Neurology	5	Other XAI methods	Quant. desc. (expert survey)	High
(5)	CP	Cell	Rule-based	Pathology	4	Without AI/XAI	Non-rand. quant. (3-condition)	High
(6)	XR	Breast	Attribution-based	Radiology	4	Without AI	Non-rand. quant. (before/after)	High
(7)	CT	Lungs	Concept; Example; Intrinsic	Radiology	6	Without XAI	Mixed-meth. (experiment + survey)	High
(8)	US	Fetus	Concept-based	Obstetrics; Sonography	46	No comparator	Quant. desc. (prospective)	Low
(9)	XR	Spine	Attribution-based	Ortho Surgery; Radiology	16	Other XAI methods	Non-rand. quant. (mixed factorial)	Moderate

Continued on next page

Ref.	Mod.	Region	XAI Category	Specialty	N	Comparator	Study Design	Risk of Bias
(10)	DM	Skin	Attribution; Example-based	Derm.; Gen. Practice	25	Other XAI; No XAI	Non-rand. quant. (multi-method)	Moderate
(11)	XR	Chest	Example-based	Radiology	10	Without XAI	Mixed-meth. (experiment + interview)	Moderate
(12)	XR	Chest	Attribution-based	Emergency Medicine	45	No comparator	Quant. desc. (prospective survey)	Low
(13)	OCT	Eye	Attribution-based	Ophthalmology	20	No comparator	Mixed-meth. (SUS + think-aloud)	Moderate
(14)	MRI	Brain	Attribution-based	Neurology	5	No comparator	Mixed-meth. (task + focus group)	High
(15)	Mixed	-	Attribution-based	Oncology; Radiology	11	Other XAI methods	Quant. desc. (survey)	High
(16)	OCT	Eye	Attribution-based	Ophthalmology	3	Other XAI methods	Quant. desc. (binary evaluation)	High
(17)	XR	Breast	Attribution-based	Radiology	1	No comparator	Quant. desc. (single expert)	High
(18)	CT	Brain	Attribution-based	Radiology	1	No comparator	Quant. desc. (single expert)	High
(19)	CT	Chest	Attribution-based	Mixed (Clin.; CS)	22	Without AI	Mixed-meth. (exploratory + survey)	High
(20)	XR	Hip	Attribution-based	Orthopaedics	-	Other XAI methods	Quant. desc. (expert survey)	High
(21)	XR	Spine	Attribution-based	Orthopaedics	16	Without XAI	Non-rand. quant. (crossover)	Moderate
(22)	DM	Skin	Intrinsic; Concept-based	Dermatology	10	No comparator	Mixed-meth. (app + feedback)	High
(23)	FP	Eye	Attribution-based	Ophthalmology	5	No comparator	Quant. desc. (blind evaluation)	High
(24)	XR	Chest	Attribution-based	Radiology	1	Other XAI methods	Quant. desc. (single expert)	High
(25)	MRI	Brain	Example-based	Neurology; Radiology	6	No comparator	Qualitative (expert interviews)	High
(26)	MRI	Heart	Example-based	Paediatric Cardiology	1	No comparator	Qualitative (expert meetings)	High
(27)	XR	Breast	Example-based	Oncology	6	No comparator	Mixed-meth. (user study + comments)	High
(28)	OCT	Eye	Attribution-based	Ophthalmology	6	Without XAI	Mixed-meth. (case evaluation)	High
(29)	XR	Lungs	Attrib.; Concept; Example	Rad.; Med.; EM	9	Without XAI	Mixed-meth. (survey + co-design)	High
(30)	CT	Brain	Attribution-based	Neurology; Radiology	9	Without AI	Non-rand. quant. (before/after)	High
(31)	DM	Skin	Attribution-based	Dermatology	2	No comparator	Quant. desc. (expert agreement)	High
(32)	MRI	Prostate	Attrib.; Uncertainty	Radiology	10	Without XAI	Mixed-meth. (thematic analysis)	Moderate
(33)	MRI	Prostate	Attribution-based	Radiology	10	Other XAI methods	Non-rand. quant. (pilot experiment)	Moderate
(34)	MRI	Prostate	Concept-based	Radiology	7	Without XAI	Non-rand. quant. (multireader)	High

Continued on next page

Ref.	Mod.	Region	XAI Category	Specialty	N	Comparator	Study Design	Risk of Bias
(35)	MRI	Pelvis	Attribution-based	Rad.; Surg.; Onc.	3	Without AI	Quant. desc. (consensus)	High
(36)	MRI	Brain	Attribution-based	General Practice	10	Other XAI methods	Mixed-meth. (experiment + qual.)	High
(37)	HP	Prostate	Attribution-based	Pathology	15	Other XAI methods	Quant. desc. (quest.)	Moderate
(38)	XR	Chest	Attribution-based	Multidisciplinary	97	Without XAI	Quant. desc. (large survey)	Low
(39)	MRI	Brain	Attribution-based	Neurosurg.; Rad.	35	Without AI/XAI	Non-rand. quant. (within-subject)	Low
(40)	MRI	Prostate	Uncertainty-based	Radiology	10	Other XAI methods	Non-rand. quant. (pilot study)	Moderate
(41)	PH	Face	Attribution-based	Clinical Genetics	31	Without XAI	Quant. desc. (clinical study)	Low
(42)	XR	Chest	Example-based	Radiology	1	No comparator	Quant. desc. (single expert)	High
(43)	EN	GI tract	Concept-based	Gastroenterology	5	Other XAI methods	Qualitative (expert evaluation)	High
(44)	XR	Chest	Example-based	Patients (Non-expert)	118	Other XAI methods	Non-rand. quant. (user study)	Low
(45)	US	Carotid	Attribution; Intrinsic	Radiology	10	Without AI	Non-rand. quant. (multicenter)	Moderate
(46)	XR	Chest	Attribution-based	Radiology	5	No comparator	Quant. desc. (eye-tracking)	High
(47)	XR; EN	Chest; Ear	Attribution-based	Otolaryngology	6	Other XAI methods	Mixed-meth. (interactive study)	High
(48)	MRI	Brain	Attribution-based	General Medicine	15	Other XAI methods	Quant. desc. (survey + scoring)	High
(49)	US; XR	Breast	Attribution-based	Radiology; Oncology	28	Other XAI methods	Non-rand. quant. (experiment)	Low
(50)	OCT	Eye	Attribution-based	Ophth.; Oncology	27	Other XAI methods	Quant. desc. (quest.)	Low
(51)	OCT	Eye	Attribution-based	Ophthalmology	3	Other XAI methods	Mixed-meth. (metrics + rating)	High

Nomenclature: XR: X-ray (incl. mammography); **CT**: Computed Tomography; **MRI**: Magnetic Resonance Imaging; **US**: Ultrasound; **OCT**: Optical Coherence Tomography; **DM**: Dermoscopy; **EN**: Endoscopy; **FP**: Fundus Photography; **HP**: Histopathology; **PH**: Photography; **CP**: Cytopathology.

3 CORPUS-LEVEL STATISTCS

The included corpus combines different clinical tasks, imaging modalities, XAI families, and evaluation architectures. Comparator arms are mixed (*no comparator*, *without XAI*, *without AI*, and *head-to-head XAI*), and primary endpoints are not harmonised (diagnostic accuracy, reading time, explanation agreement, preference, trust, usability, etc., on different instruments). Aggregating these into a single numeric synthesis would imply exchangeability of effects that the studies do not support. We therefore report **corpus-level descriptive statistics** as the appropriate quantitative complement to narrative synthesis.

Table S3. Imaging modality frequency in included papers

Imaging modality (as coded in extraction)	n
XR / chest radiography (XR)	17
MRI	12
OCT	5
CT	4
Dermoscopy / DM	3
Ultrasound (US)	2
Other / mixed (single modality each: CP, EN, FP, HP, PH, Mixed; one XR+EN; one US+XR)	8
Total	51

Table S4. Frequency and type of comparator used in included papers

Comparator	n
Other XAI methods	18
No comparator	15
Without XAI	10
Without AI	5
Without AI; Without XAI	2
Other XAI methods; Without XAI	1
Total	51

4 IMAGING MODALITY

4.1 Anatomical focus

The largest anatomical groupings include chest imaging and lung tasks combined (**13**/51), brain (**8**/51), eye (**6**/51), breast (**4**/51), and prostate (**5**/51, of which **4**/51 are MRI studies of prostate lesion detection with radiology readers: (32); (33); (34); (40)). These clusters support structured narrative comparison of similar evaluation questions; they do not justify pooled inference without harmonised endpoints.

REFERENCES

[1]Bergomi L, Nicora G, Orlowska MA, Podrecca C, Bellazzi R, Fregosi C, et al. Which explanations do clinicians prefer? A comparative evaluation of XAI understandability and actionability in predicting the need for hospitalization. BMC Medical Informatics and Decision Making. 2025;25(1):269.

[2]Cabitza F, Natali C, Famiglini L, Campagner A, Caccavella V, Gallazzi E. Never tell me the odds: Investigating pro-hoc explanations in medical decision making. Artificial intelligence in medicine. 2024;150:102819.

[3]Min H, You T, Lee H, Cho Y, Cho S. InstructX2X: An Interpretable Local Editing Model for Counterfactual Medical Image Generation. In: International Conference on Medical Image Computing and Computer-Assisted Intervention. Springer; 2025. p. 279-88.

[4]Al-Bakri FH, Bejuri WMYW, Al-Andoli MN, Ikram RRR, Khor HM, Sholva Y, et al. A Feature-Augmented Explainable Artificial Intelligence Model for Diagnosing Alzheimer's Disease from Multimodal Clinical and Neuroimaging Data. Diagnostics. 2025;15(16):2060.

[5]Chen H, Gomez C, Correa ZM, Liu A, Milman T, Eiger-Moscovich M, et al. An interactive and explainable AI approach to improve human-machine teaming in cancer subtyping from digital cytopathology. Medical image analysis. 2025:103856.

[6]Jungmann F, Ziegelmayer S, Lohoefer FK, Metz S, Müller-Leisse C, Englmaier M, et al. Algorithmic transparency and interpretability measures improve radiologists' performance in BI-RADS 4 classification. European Radiology. 2023;33(3):1844-51.

[7]Gallée L, Lisson CS, Lisson CG, Drees D, Weig F, Vogele D, et al. Evaluating the explainability of attributes and prototypes for a medical classification model. In: World Conference on Explainable Artificial Intelligence. Springer; 2024. p. 43-56.

[8]Bashir Z, Lin M, Feragen A, Mikolaj K, Taksøe-Vester C, Christensen AN, et al. Clinical validation of explainable AI for fetal growth scans through multi-level, cross-institutional prospective end-user evaluation. Scientific Reports. 2025;15(1):2074.

[9]Famiglini L, Campagner A, Barandas M, La Maida GA, Gallazzi E, Cabitza F. Evidence-based XAI: An empirical approach to design more effective and explainable decision support systems. Computers in biology and medicine. 2024;170:108042.

[10]Buijing B, Sent D. Exploring Differential Diagnosis-Based Explainable AI: A Case Study in Melanoma Detection. Studies in health technology and informatics. 2025;327:507-11.

[11]Cabitza F, Famiglini L, Fregosi C, Pe S, Parimbelli E, La Maida GA, et al. From oracular to judicial: enhancing clinical decision making through contrasting explanations and a novel interaction protocol. In: Proceedings of the 30th international conference on intelligent user interfaces; 2025. p. 745-54.

[12]Carlile M, Hurt B, Hsiao A, Hogarth M, Longhurst CA, Dameff C. Deployment of artificial intelligence for radiographic diagnosis of COVID-19 pneumonia in the emergency department. JACEP Open. 2020;1(6):1459-64.

[13]Chen JS, Baxter SL, van den Brandt A, Lieu A, Camp AS, Do JL, et al. Usability and clinician acceptance of a deep learning-based clinical decision support tool for predicting glaucomatous visual field progression. Journal of glaucoma. 2023;32(3):151-8.

[14]de Bonis MLN, Fasano G, Lombardi A, Testino A, di Sciascio E, di Noia T, et al. Explainable AI for Brain Age Prediction: Design, Implementation, and Formative Evaluation of an Interactive Tool. In: HHAI 2025. IOS Press; 2025. p. 248-61.

[15]Dominguez D, Fotopoulos D, Chouvarda I, Ausso S. Designing a Method to Identify Explainability Requirements in Cancer Research. In: Joint European Conference on Machine Learning and Knowledge Discovery in Databases. Springer; 2023. p. 382-8.

[16]Elsawy A, Keenan TD, Chen Q, Shi X, Thavikulwat AT, Bhandari S, et al. Deep-GA-Net for accurate and explainable detection of geographic atrophy on OCT scans. Ophthalmology Science. 2023;3(4):100311.

[17]Ergün U, Çoban T, Kayadibi İ. BCECNN: an explainable deep ensemble architecture for accurate diagnosis of breast cancer. BMC Medical Informatics and Decision Making. 2025;25(1):374.

[18]Highton J, Chong QZ, Crawley R, Schnabel JA, Bhatia KK. Evaluation of randomized input sampling for explanation (RISE) for 3D XAI-proof of concept for black-box brain-hemorrhage classification. In: International Conference on Medical Imaging and Computer-Aided Diagnosis. Springer; 2023. p. 41-51.

[19]Feng J, Rahrooh A, Bui A. Error Profiling of Machine Learning Models: An Exploratory Visualization. In: Machine Learning for Healthcare Conference. PMLR; 2025. .

[20]Feng SW, Lin SY, Shen YT, Chen BY, Chen YC, Chiang YH. Sensor-based Explainable Deep Learning Framework for Osteoporosis Screening via Hip X-ray Interpretation. Sensors & Materials. 2025;37.

[21]Natali C, Famiglini L, Campagner A, La Maida GA, Gallazzi E, Cabitza F. Color shadows 2: Assessing the impact of xai on diagnostic decision-making. In: World Conference on Explainable Artificial Intelligence. Springer; 2023. p. 618-29.

[22]Gareau DS, Browning J, Correa Da Rosa J, Suarez-Farinas M, Lish S, Zong AM, et al. Deep learning-level melanoma detection by interpretable machine learning and imaging biomarker cues. Journal of Biomedical Optics. 2020;25(11):112906-6.

[23]Niu Y, Gu L, Lu F, Lv F, Wang Z, Sato I, et al. Pathological evidence exploration in deep retinal image diagnosis. In: Proceedings of the AAAI conference on artificial intelligence. vol. 33; 2019. p. 1093-101.

[24]Kinger S, Kulkarni V. Transparent and trustworthy interpretation of COVID-19 features in chest X-rays using explainable AI. Multimedia Tools and Applications. 2025;84(18):19853-81.

[25]Singh D, Brima Y, Levin F, Becker M, Hiller B, Hermann A, et al. An unsupervised XAI framework for dementia detection with context enrichment. Scientific reports. 2025;15(1):39554.

[26]Guo G, Deng L, Tandon A, Endert A, Kwon BC. Mimicri: Towards domain-centered counterfactual explanations of cardiovascular image classification models. In: Proceedings of the 2024 ACM Conference on Fairness, Accountability, and Transparency; 2024. p. 1861-74.

[27]Lamy JB, Sekar B, Guezennec G, Bouaud J, Séroussi B. Explainable artificial intelligence for breast cancer: A visual case-based reasoning approach. Artificial intelligence in medicine. 2019;94:42-53.

[28]van den Brandt A, Christopher M, Zangwill LM, Rezapour J, Bowd C, Baxter SL, et al. GLANCE: Visual Analytics for Monitoring Glaucoma Progression. In: VCBM; 2020. p. 85-96.

[29]Xie Y, Chen M, Kao D, Gao G, Chen X. CheXplain: enabling physicians to explore and understand data-driven, AI-enabled medical imaging analysis. In: Proceedings of the 2020 CHI Conference on Human Factors in Computing Systems; 2020. p. 1-13.

[30]Yang KC, Xu Y, Lin Q, Tang LL, Zhong Jw, An HN, et al. Explainable deep learning algorithm for identifying cerebral venous sinus thrombosis-related hemorrhage (CVST-ICH) from spontaneous intracerebral hemorrhage using computed tomography. EClinicalMedicine. 2025;81.

[31]Giavina-Bianchi M, Vitor WG, Fornasiero de Paiva V, Okita AL, Sousa RM, Machado B. Explainability agreement between dermatologists and five visual explanations techniques in deep neural networks for melanoma AI classification. Frontiers in Medicine. 2023;10:1241484.

[32]Gulum MA, Trombley CM, Ozen M, Esen E, Aksamoglu M, Kantardzic M. Why are explainable AI methods for prostate lesion detection rated poorly by radiologists? Applied Sciences. 2024;14(11):4654.

[33]Gulum MA, Trombley CM, Ozen M, Kantardzic M. Are Post-Hoc Explanation Methods for Prostate Lesion Detection Effective for Radiology End Use? In: 2022 21st IEEE International Conference on Machine Learning and Applications (ICMLA). IEEE; 2022. p. 1183-9.

[34]Hamm CA, Baumgärtner GL, Biessmann F, Beetz NL, Hartenstein A, Savic LJ, et al. Interactive explainable deep learning model informs prostate cancer diagnosis at MRI. Radiology. 2023;307(4):e222276.

[35]Kobayashi K, Takamizawa Y, Miyake M, Ito S, Gu L, Nakatsuka T, et al. Can physician judgment enhance model trustworthiness? A case study on predicting pathological lymph nodes in rectal cancer. Artificial Intelligence in Medicine. 2024;154:102929.

[36]Kumar A, Manikandan R, Kose U, Gupta D, Satapathy SC. Doctor's dilemma: evaluating an explainable subtractive spatial lightweight convolutional neural network for brain tumor diagnosis. ACM Transactions on Multimedia Computing, Communications, and Applications (TOMM). 2021;17(3s):1-26.

[37]Manz R, Baecker J, Cramer S, Meyer P, Müller D, Muzalyova A, et al. Do explainable AI (XAI) methods improve the acceptance of AI in clinical practice? An evaluation of XAI methods on Gleason grading. The Journal of Pathology: Clinical Research. 2025;11(2):e70023.

[38]Karagoz G, van Kollenburg G, Ozcelebi T, Meratnia N. Evaluating How Explainable AI Is Perceived in the Medical Domain: A Human-Centered Quantitative Study of XAI in Chest X-Ray Diagnostics. In: International Workshop on Trustworthy Artificial Intelligence for Healthcare. Springer; 2024. p. 92-108.
[39]Jin W, Fatehi M, Guo R, Hamarneh G. Evaluating the clinical utility of artificial intelligence assistance and its explanation on the glioma grading task. Artificial Intelligence in Medicine. 2024;148:102751.
[40]Trombley CM, Gulum MA, Ozen M, Esen E, Aksamoglu M, Kantardzic M. Evaluating uncertainty-based deep learning explanations for prostate lesion detection. In: Machine Learning for Healthcare Conference. PMLR; 2022. p. 874-91.
[41]Sümer Ö, Huber T, Duong D, Hanchard SEL, Conati C, André E, et al. Evaluation of a Deep Learning and XAI based Facial Phenotyping Tool for Genetic Syndromes: A Clinical User Study. medRxiv. 2025.
[42]Yazdani E, Neizehbaz A, Karamzade-Ziarati N, Kheradpisheh SR. Explainable artificial intelligence for pneumonia classification: clinical insights into deformable prototypical part network in pediatric chest x-ray images. Journal of medical imaging and radiation sciences. 2025;56(5):102023.
[43]Storås AM, Dreyer M, Pahde F, Lapuschkin S, Samek W, Halvorsen P, et al. Exploring the clinical value of concept-based AI explanations in gastrointestinal disease detection. Scientific Reports. 2025;15(1):28860.
[44]Mertes S, Huber T, Weitz K, Heimerl A, André E. Ganterfactual—counterfactual explanations for medical non-experts using generative adversarial learning. Frontiers in artificial intelligence. 2022;5:825565.
[45]Liu J, Zhou X, Lin H, Huang Y, Zheng J, Xu E, et al. Human–machine interaction based on real-time explainable deep learning for higher accurate grading of carotid stenosis from transverse B-mode scan videos. European Journal of Radiology. 2025:112441.
[46]Pham TT, Brecheisen J, Wu CC, Nguyen H, Deng Z, Adjeroh D, et al. ItpCtrl-AI: End-to-end interpretable and controllable artificial intelligence by modeling radiologists' intentions. Artificial intelligence in medicine. 2025;160:103054.
[47]Shi S, Shao Y, Jiang H, Yao Y, Zhang Z, Ding X, et al. MEDebiaser: A Human-AI Feedback System for Mitigating Bias in Multi-label Medical Image Classification. In: Proceedings of the 38th Annual ACM Symposium on User Interface Software and Technology; 2025. p. 1-27.
[48]Marmolejo-Saucedo JA, Kose U. Numerical grad-cam based explainable convolutional neural network for brain tumor diagnosis. Mobile Networks and Applications. 2024;29(1):109-18.
[49]Rezaeian O, Asan O, Bayrak AE. The impact of AI explanations on clinicians' trust and diagnostic accuracy in breast cancer. Applied Ergonomics. 2025;129:104577.
[50]Wysocki O, Mak S, Frost H, Graham DM, Landers D, Aslam T. Translating the machine; An assessment of clinician understanding of ophthalmological artificial intelligence outputs. International journal of medical informatics. 2025;201:105958.
[51]Singh A, Sengupta S, Mohammed AR, Faruq I, Jayakumar V, Zelek J, et al. What is the optimal attribution method for explainable ophthalmic disease classification? In: International Workshop on Ophthalmic Medical Image Analysis. Springer; 2020. p. 21-31.
