# Supplementary material for: Which explainable AI methods in medical imaging are clinically impactful? A systematic literature review addressing the clinician's perspective
Source: Front Artif Intell. 2026 May 29;9:1819422. doi: 10.3389/frai.2026.1819422 (PMC13260647; doi:10.3389/frai.2026.1819422)
Supplement: Supplementary file 4 [file Supplementary_file_4.pdf]

# Supplementary Material

## 1 CLAUDE.MD (SCREENING LOGIC AND DATA STRUCTURE)

```
# SLR Screening Rules: XAI in Medical Imaging

## Inclusion Criteria (ALL must be true)

1. Uses any machine learning technique
2. Uses medical images (any modality)
3. Includes at least one explainability/interpretability method
4. Involves user (clinician/patient) participation in evaluation
5. Is primary research only
6. Multimodal methods are acceptable (single modality is also fine)

## Exclusion Criteria (ANY one is sufficient to exclude)

- Reviews
- Surveys
- No radiologist/healthcare professional involvement
- Editorials, opinion pieces, or review papers
- EEG-based studies
- Not used for clinical decision making
- Case studies
- Animal studies
- Other than English language
- No explainability method

## Screening Logic

A paper is Included only if ALL inclusion criteria are satisfied AND none of
    the exclusion criteria apply.

## Dataset Structure: SLR Data Extraction CSV

### Column Structure (48 Columns)

1-6: Basic Information (Reviewer, Title, DOI, Decision, etc.)
7-13: Image and Dataset (Modality, Anatomical Region, Task, etc.)
14-21: Machine Learning Model (Architecture, Setup, Granularity, etc.)
22-29: XAI Methods (Category, Scope, Output Format, Timing, etc.)
30-37: Clinical Evaluation Setup (Clinician Count, Specialty, Exp Type, etc
   .)
```

```
38-45: Assessment Outcomes (Trust, Decision Impact, Actionability, etc.)
46-48: Additional Information (Limitations, Design, Comments)
```

### 1.1 AI Agent Instruction (Prompt)

The following prompt was used to direct the AI assistant (Claude) to perform the screening and data extraction tasks in parallel using the rules defined above.

```
Read the next 10 papers from the csv list that aren't reviewed yet, decide
   if the paper should be included or excluded and update the spreadsheet
   with all relevant columns and add yourself (claude) as the reviewer for
   that row.

You should read the papers from the local folder first. Fetching the DOI is
   usually behind a paywall and won't work. Please read and evaluate each
   paper in its own subagent to save on your own context space.

Please create tasks (todos) for rating the remaining papers in the csv and
   updating the csv, always do 10 papers in parallel using subagents per
   task.
```

## 2 INTER-RATER AGREEMENT

### 2.1 Abstract Screening

**Table S1.** Pairwise screening agreement between human reviewers and the automated abstract screener (Claude).

| Annotator 1 | Annotator 2 | $N$ | Agree | Agreement (%) |
|---|---|---|---|---|
| SUD | Claude | 3348 | 3089 | 92.26 |
| EB | Claude | 1053 | 1022 | 97.06 |
| AL | Claude | 919 | 787 | 85.64 |
| MI | Claude | 451 | 440 | 97.56 |
| AL | SUD | 63 | 54 | 85.71 |
| AL | MI | 11 | 6 | 54.55 |
| SUD | MI | 7 | 7 | 100.00 |
| AL | EB | 3 | 0 | 0.00 |
| SUD | EB | 2 | 0 | 0.00 |
| MI | EB | — | — | — |

Agreement was assessed on the merged Rayyan export, one row per bibliographic record ($N_{\text{corpus}} = 5687$). For each unordered pair of annotators, we selected all rows where *both* members of the pair had a non-empty screening label on that row. The automated screener (reported as "Claude") contains only *Included* or *Excluded* (rule-based screening of title and abstract text). Human reviewers could assign *Included*, *Excluded*, or *Maybe*. **Agreement** was defined as an *exact* match between the two labels on the same row. Thus, *Maybe* was *not* collapsed into Included or Excluded: e.g. *Included* vs. *Maybe* counts as disagreement, as does *Maybe* vs.Excluded. Any human *Maybe* vs. Claude *Included*/*Excluded* is therefore counted as disagreement. The table reports $N$ (jointly labelled rows), the number of agreeing rows, the percentage

agreement, and sorts pairs by decreasing $N$. Cohen's $\kappa$ was not used in this table (high prevalence of excluded labels makes $\kappa$ easy to misread alongside percent agreement).

Across human reviewers, the majority of decisions were *Excluded* (exclusion rates in this initial screening were ranging between 90% -99% papers). High percent agreement therefore partly reflects shared exclusion on clearly out-of-scope items, not only deep consensus on borderline work.

On the relatively small set of records where AJ and SUD both screened ($N = 63$), most decisions matched; the nine mismatches were asymmetric: in eight cases AJ recorded *Excluded* while SUD recorded *Included* or *Maybe*; in one case AJ recorded *Included* while SUD recorded *Excluded*. Thus, among their disagreements, AJ more often took the stricter (exclusion) side. The AJ – EB and SUD – EB pairs had very small $N$ (3 and 2 joint records); agreement was 0% in those cells and should be interpreted cautiously. MI and EB never appeared together ($N = 0$).

#### Quantitative comparison (Claude – human vs human – human).

Exact-match **weighted** percent agreement:

- Human – human (all pairs with $N > 0$): 77.9% ($N = 86$).
- Human – human (pairs with $N \geq 10$): 81.1% ($N = 74$).
- Claude – human (four pairs): 92.5%($N = 5771$).

Thus, on a weighted basis, Claude – human exact agreement exceeds pooled human – human agreement, but the denominators and overlap structure differs; the restricted human – human baselines narrow the gap (e.g. 85.7% for AJ – SUD ($N = 63$) vs 85.6% for AJ – Claude ($N = 919$)).

Disagreements with Claude are dominated by inclusion/exclusion threshold differences on borderline abstracts, and by human use of *Maybe*.

### 2.2 Full Text Screening

**Table S2.** Claude vs human reviewers (AL and SUD): Full Text Screening.

| Comparison | $N$ | Exact agreement | Cohen's $\kappa$ |
|---|---|---|---|
| Claude vs AL | 64 | 82.8% | 0.60 |
| Claude vs SUD | 59 | 89.8% | 0.71 |

On full screened papers by humans and Claude, Claude endorsed inclusion more often than either human:

- **vs SUD:** SUD 10/59 included (16.9%), Claude 16/59 (27.1%)—6 more inclusions by Claude.
- **vs AL:** AL 18/64 included (28.1%), Claude 21/64 (32.8%)—3 more inclusions by Claude.

Disagreements were therefore asymmetric, with several records excluded by the human but Included by Claude. All papers included by Claude were subsequently reviewed by SUD, and data extraction was corrected where necessary. Full texts that were not initially reviewed by a human reviewer but were excluded by Claude were double-checked by M.I. to verify the reason for exclusion.

# 3 INTER-RATER AGREEMENT

## 3.1 Abstract Screening

**Table S3.** Pairwise screening agreement between human reviewers and the automated abstract screener (Claude).

| Annotator 1 | Annotator 2 | $N$ | Agree | Agreement (%) |
|---|---|---|---|---|
| SUD | Claude | 3348 | 3089 | 92.26 |
| EB | Claude | 1053 | 1022 | 97.06 |
| AL | Claude | 919 | 787 | 85.64 |
| MI | Claude | 451 | 440 | 97.56 |
| AL | SUD | 63 | 54 | 85.71 |
| AL | MI | 11 | 6 | 54.55 |
| SUD | MI | 7 | 7 | 100.00 |
| AL | EB | 3 | 0 | 0.00 |
| SUD | EB | 2 | 0 | 0.00 |
| MI | EB | — | — | — |

Agreement was assessed on the merged Rayyan export, one row per bibliographic record ($N_{\text{corpus}} = 5687$). For each unordered pair of annotators, we selected all rows where *both* members of the pair had a non-empty screening label on that row. The automated screener (reported as "Claude") contains only *Included* or *Excluded* (rule-based screening of title and abstract text). Human reviewers could assign *Included*, *Excluded*, or *Maybe*. **Agreement** was defined as an *exact* match between the two labels on the same row. Thus, *Maybe* was *not* collapsed into Included or Excluded: e.g. *Included* vs. *Maybe* counts as disagreement, as does *Maybe* vs.Excluded. Any human *Maybe* vs. Claude *Included/Excluded* is therefore counted as disagreement. The table reports $N$ (jointly labelled rows), the number of agreeing rows, the percentage agreement, and sorts pairs by decreasing $N$. Cohen's $\kappa$ was not used in this table (high prevalence of excluded labels makes $\kappa$ easy to misread alongside percent agreement).

Across human reviewers, the majority of decisions were *Excluded* (exclusion rates in this initial screening were ranging between 90% -99% papers). High percent agreement therefore partly reflects shared exclusion on clearly out-of-scope items, not only deep consensus on borderline work.

On the relatively small set of records where AJ and SUD both screened ($N = 63$), most decisions matched; the nine mismatches were asymmetric: in eight cases AJ recorded *Excluded* while SUD recorded *Included* or *Maybe*; in one case AJ recorded *Included* while SUD recorded *Excluded*. Thus, among their disagreements, AJ more often took the stricter (exclusion) side. The AJ – EB and SUD – EB pairs had very small $N$ (3 and 2 joint records); agreement was 0% in those cells and should be interpreted cautiously. MI and EB never appeared together ($N = 0$).

### *Quantitative comparison (Claude – human vs human – human).*

Exact-match **weighted** percent agreement:

- Human – human (all pairs with $N > 0$): 77.9% ($N = 86$).
- Human – human (pairs with $N \geq 10$): 81.1% ($N = 74$).
- Claude – human (four pairs): 92.5%($N = 5771$).

Thus, on a weighted basis, Claude – human exact agreement exceeds pooled human – human agreement, but the denominators and overlap structure differs; the restricted human – human baselines narrow the gap (e.g. 85.7% for AJ – SUD ($N = 63$) vs 85.6% for AJ – Claude ($N = 919$)).

Disagreements with Claude are dominated by inclusion/exclusion threshold differences on borderline abstracts, and by human use of *Maybe*.

### 3.2 Full Text Screening

**Table S4.** Claude vs human reviewers (AL and SUD): Full Text Screening.

| Comparison | $N$ | Exact agreement | Cohen's $\kappa$ |
|---|---|---|---|
| Claude vs AL | 64 | 82.8% | 0.60 |
| Claude vs SUD | 59 | 89.8% | 0.71 |

On full screened papers by humans and Claude, Claude endorsed inclusion more often than either human:

- **vs SUD:** SUD 10/59 included (16.9%), Claude 16/59 (27.1%)—6 more inclusions by Claude.
- **vs AL:** AL 18/64 included (28.1%), Claude 21/64 (32.8%)—3 more inclusions by Claude.

Disagreements were therefore asymmetric, with several records excluded by the human but Included by Claude. All papers included by Claude were subsequently reviewed by SUD, and data extraction was corrected where necessary. Full texts that were not initially reviewed by a human reviewer but were excluded by Claude were double-checked by M.I. to verify the reason for exclusion.
