# Supplementary material for: Which explainable AI methods in medical imaging are clinically impactful? A systematic literature review addressing the clinician's perspective
Source: Front Artif Intell. 2026 May 29;9:1819422. doi: 10.3389/frai.2026.1819422 (PMC13260647; doi:10.3389/frai.2026.1819422)
Supplement: Supplementary file 5 [file Supplementary_file_5.pdf]

frontiers

Supplementary Material

1 RISK OF BIAS

Table S1. MMAT Quality Criteria by Study Design Category

Cat.	C#	Criterion
Qualitative	C1	Is the qualitative approach appropriate to answer the research question?
	C2	Are the qualitative data collection methods adequate?
	C3	Are the findings adequately derived from the data?
	C4	Is the interpretation of results sufficiently substantiated by data?
	C5	Is there coherence between data sources, collection, analysis, and interpretation?
Non-rand. quant.	C1	Are the participants representative of the target population?
	C2	Are measurements appropriate regarding outcome and intervention?
	C3	Are there complete outcome data?
	C4	Are the confounders accounted for in the design and analysis?
	C5	Was the intervention administered as intended?
Quant. desc.	C1	Is the sampling strategy relevant to address the research question?
	C2	Is the sample representative of the target population?
	C3	Are the measurements appropriate?
	C4	Is the risk of nonresponse bias low?
	C5	Is the statistical analysis appropriate?
Mixed-methods	C1	Is there an adequate rationale for using a mixed methods design?
	C2	Are the different components effectively integrated?
	C3	Are the outputs of integration adequately interpreted?
	C4	Are divergences between quantitative and qualitative results addressed?
	C5	Do the different components adhere to the quality criteria of each tradition?

Table S2: MMAT Risk of Bias Assessment for All Included Studies. The MMAT column shows the original rating based on standard MMAT scoring; the Adj. column shows the rating after sample-size and study-scope adjustment.

Ref.	Design	C1	C2	C3	C4	C5	Y	N	MMAT	Adj.
(1)	MM	Y	Y	Y	CT	CT	3	0	Mod	Mod
(2)	NRQ	Y	Y	Y	CT	Y	4	0	Low	Mod
(3)	QD	CT	N	Y	CT	CT	1	1	High	High
(4)	QD	CT	N	Y	CT	CT	1	1	High	High
(5)	NRQ	CT	Y	Y	CT	Y	3	0	Mod	High
(6)	NRQ	Y	Y	Y	CT	Y	4	0	Low	High
(7)	MM	Y	Y	Y	CT	CT	3	0	Mod	High
(8)	QD	Y	Y	Y	Y	Y	5	0	Low	Low
(9)	NRQ	Y	Y	Y	Y	Y	5	0	Low	Mod
(10)	NRQ	Y	Y	CT	CT	Y	3	0	Mod	Mod
(11)	MM	Y	Y	Y	CT	Y	4	0	Low	Mod
(12)	QD	Y	Y	Y	CT	Y	4	0	Low	Low
(13)	MM	Y	Y	Y	CT	CT	3	0	Mod	Mod
(14)	MM	Y	Y	Y	CT	CT	3	0	Mod	High
(15)	QD	CT	CT	Y	CT	CT	1	0	High	High
(16)	QD	CT	N	Y	CT	CT	1	1	High	High
(17)	QD	N	N	CT	N	N	0	4	High	High

Continued on next page

Ref.	Design	C1	C2	C3	C4	C5	Y	N	MMAT	Adj.
(18)	QD	N	N	Y	N	N	1	4	High	High
(19)	MM	Y	CT	CT	CT	CT	1	0	High	High
(20)	QD	CT	CT	Y	CT	CT	1	0	High	High
(21)	NRQ	Y	Y	Y	Y	Y	5	0	Low	Mod
(22)	MM	CT	CT	CT	CT	N	0	1	High	High
(23)	QD	CT	CT	Y	CT	Y	2	0	High	High
(24)	QD	N	N	Y	N	CT	1	3	High	High
(25)	QL	Y	CT	Y	Y	CT	3	0	Mod	High
(26)	QL	Y	CT	Y	Y	N	3	1	High	High
(27)	MM	CT	CT	CT	CT	CT	0	0	High	High
(28)	MM	Y	Y	Y	CT	CT	3	0	Mod	High
(29)	MM	Y	Y	Y	CT	CT	3	0	Mod	High
(30)	NRQ	Y	Y	Y	Y	Y	5	0	Low	High
(31)	QD	CT	N	Y	CT	Y	2	1	High	High
(32)	MM	Y	Y	Y	Y	CT	4	0	Low	Mod
(33)	NRQ	Y	Y	Y	CT	Y	4	0	Low	Mod
(34)	NRQ	Y	Y	Y	Y	Y	5	0	Low	High
(35)	QD	CT	N	Y	CT	Y	2	1	High	High
(36)	MM	CT	Y	Y	CT	CT	2	0	High	High
(37)	QD	Y	Y	Y	CT	Y	4	0	Low	Mod
(38)	QD	Y	Y	Y	CT	Y	4	0	Low	Low
(39)	NRQ	Y	Y	Y	Y	Y	5	0	Low	Low
(40)	NRQ	Y	Y	Y	CT	Y	4	0	Low	Mod
(41)	QD	Y	Y	Y	CT	Y	4	0	Low	Low
(42)	QD	N	N	Y	N	N	1	4	High	High
(43)	QD	CT	N	Y	CT	CT	1	1	High	High
(44)	NRQ	Y	Y	Y	CT	Y	4	0	Low	Low
(45)	NRQ	Y	Y	Y	Y	Y	5	0	Low	Mod
(46)	QD	CT	CT	Y	CT	Y	2	0	High	High
(47)	MM	CT	CT	CT	CT	CT	0	0	High	High
(48)	QD	CT	CT	Y	CT	Y	2	0	High	High
(49)	NRQ	Y	Y	Y	CT	Y	4	0	Low	Low
(50)	QD	Y	Y	Y	CT	Y	4	0	Low	Low
(51)	MM	CT	Y	Y	CT	CT	2	0	High	High

Abbreviations: Design: QL = Qualitative, NRQ = Non-randomised quantitative, QD = Quantitative descriptive, MM = Mixed-methods. **Ratings**: Y = Yes, N = No, CT = Can't tell. **MMAT**: Original risk of bias based on standard MMAT scoring (Low = $\leq$1 N/CT; Moderate = 2 N/CT; High = $\geq$3 N/CT). **Adj.**: Risk of bias after sample-size and study-scope adjustment (studies with $N < 10$ clinicians cannot be rated lower than High; N 10–19 cannot be rated lower than Moderate).

1.1 Comparison of Initial MMAT Scores vs. Adjusted Risk of Bias

Table S3. Comparison of Initial MMAT Scores vs. Adjusted Risk of Bias (N=51)

Risk Category	MMAT (Initial)	After Adjustment
Low Risk	20	8
Moderate Risk	9	12
High Risk	22	31

The adjustment was quite strict, it shifted many papers upward in risk:

- Nine studies were downgraded from Low Risk to Moderate due to small sample sizes (N = 10–16):(2; 9; 11; 21; 32; 33; 37; 40; 45).
- Three studies were downgraded from Low to High despite scoring well on standard MMAT criteria, owing to very small clinician samples (N = 4–9) or single-centre designs: (6; 30; 34).
- Six studies were downgraded from Moderate to High due to very small clinician samples (N = 4–6) or limited qualitative saturation: (5; 7; 14; 25; 28; 29).
- Three studies remained at Moderate risk after adjustment: (1; 10; 13). Eight studies remained at Low risk, all characterised by relatively large sample sizes ($N \geq 27$) and/or multi-centre or prospective designs: (8; 12; 38; 39; 41; 44; 49; 50). All 22 studies originally rated High remained High after adjustment.

REFERENCES

[1]Bergomi L, Nicora G, Orlowska MA, Podrecca C, Bellazzi R, Fregosi C, et al. Which explanations do clinicians prefer? A comparative evaluation of XAI understandability and actionability in predicting the need for hospitalization. BMC Medical Informatics and Decision Making. 2025;25(1):269.

[2]Cabitza F, Natali C, Famiglini L, Campagner A, Caccavella V, Gallazzi E. Never tell me the odds: Investigating pro-hoc explanations in medical decision making. Artificial intelligence in medicine. 2024;150:102819.

[3]Min H, You T, Lee H, Cho Y, Cho S. InstructX2X: An Interpretable Local Editing Model for Counterfactual Medical Image Generation. In: International Conference on Medical Image Computing and Computer-Assisted Intervention. Springer; 2025. p. 279-88.

[4]Al-Bakri FH, Bejuri WMYW, Al-Andoli MN, Ikram RRR, Khor HM, Sholva Y, et al. A Feature-Augmented Explainable Artificial Intelligence Model for Diagnosing Alzheimer's Disease from Multimodal Clinical and Neuroimaging Data. Diagnostics. 2025;15(16):2060.

[5]Chen H, Gomez C, Correa ZM, Liu A, Milman T, Eiger-Moscovich M, et al. An interactive and explainable AI approach to improve human-machine teaming in cancer subtyping from digital cytopathology. Medical image analysis. 2025:103856.

[6]Jungmann F, Ziegelmayer S, Lohoefer FK, Metz S, Müller-Leisse C, Englmaier M, et al. Algorithmic transparency and interpretability measures improve radiologists' performance in BI-RADS 4 classification. European Radiology. 2023;33(3):1844-51.

[7]Gallée L, Lisson CS, Lisson CG, Drees D, Weig F, Vogele D, et al. Evaluating the explainability of attributes and prototypes for a medical classification model. In: World Conference on Explainable Artificial Intelligence. Springer; 2024. p. 43-56.

[8]Bashir Z, Lin M, Feragen A, Mikolaj K, Taksøe-Vester C, Christensen AN, et al. Clinical validation of explainable AI for fetal growth scans through multi-level, cross-institutional prospective end-user evaluation. Scientific Reports. 2025;15(1):2074.

[9]Famiglini L, Campagner A, Barandas M, La Maida GA, Gallazzi E, Cabitza F. Evidence-based XAI: An empirical approach to design more effective and explainable decision support systems. Computers in biology and medicine. 2024;170:108042.

[10]Buijing B, Sent D. Exploring Differential Diagnosis-Based Explainable AI: A Case Study in Melanoma Detection. Studies in health technology and informatics. 2025;327:507-11.

[11]Cabitza F, Famiglini L, Fregosi C, Pe S, Parimbelli E, La Maida GA, et al. From oracular to judicial: enhancing clinical decision making through contrasting explanations and a novel interaction protocol. In: Proceedings of the 30th international conference on intelligent user interfaces; 2025. p. 745-54.

[12]Carlile M, Hurt B, Hsiao A, Hogarth M, Longhurst CA, Dameff C. Deployment of artificial intelligence for radiographic diagnosis of COVID-19 pneumonia in the emergency department. JACEP Open. 2020;1(6):1459-64.

[13]Chen JS, Baxter SL, van den Brandt A, Lieu A, Camp AS, Do JL, et al. Usability and clinician acceptance of a deep learning-based clinical decision support tool for predicting glaucomatous visual field progression. Journal of glaucoma. 2023;32(3):151-8.

[14]de Bonis MLN, Fasano G, Lombardi A, Testino A, di Sciascio E, di Noia T, et al. Explainable AI for Brain Age Prediction: Design, Implementation, and Formative Evaluation of an Interactive Tool. In: HHAI 2025. IOS Press; 2025. p. 248-61.

[15]Dominguez D, Fotopoulos D, Chouvarda I, Ausso S. Designing a Method to Identify Explainability Requirements in Cancer Research. In: Joint European Conference on Machine Learning and Knowledge Discovery in Databases. Springer; 2023. p. 382-8.

[16]Elsawy A, Keenan TD, Chen Q, Shi X, Thavikulwat AT, Bhandari S, et al. Deep-GA-Net for accurate and explainable detection of geographic atrophy on OCT scans. Ophthalmology Science. 2023;3(4):100311.

[17]Ergün U, Çoban T, Kayadibi İ. BCECNN: an explainable deep ensemble architecture for accurate diagnosis of breast cancer. BMC Medical Informatics and Decision Making. 2025;25(1):374.

[18]Highton J, Chong QZ, Crawley R, Schnabel JA, Bhatia KK. Evaluation of randomized input sampling for explanation (RISE) for 3D XAI-proof of concept for black-box brain-hemorrhage classification. In: International Conference on Medical Imaging and Computer-Aided Diagnosis. Springer; 2023. p. 41-51.

[19]Feng J, Rahrooh A, Bui A. Error Profiling of Machine Learning Models: An Exploratory Visualization. In: Machine Learning for Healthcare Conference. PMLR; 2025. .

[20]Feng SW, Lin SY, Shen YT, Chen BY, Chen YC, Chiang YH. Sensor-based Explainable Deep Learning Framework for Osteoporosis Screening via Hip X-ray Interpretation. Sensors & Materials. 2025;37.

[21]Natali C, Famiglini L, Campagner A, La Maida GA, Gallazzi E, Cabitza F. Color shadows 2: Assessing the impact of xai on diagnostic decision-making. In: World Conference on Explainable Artificial Intelligence. Springer; 2023. p. 618-29.

[22]Gareau DS, Browning J, Correa Da Rosa J, Suarez-Farinas M, Lish S, Zong AM, et al. Deep learning-level melanoma detection by interpretable machine learning and imaging biomarker cues. Journal of Biomedical Optics. 2020;25(11):112906-6.

[23]Niu Y, Gu L, Lu F, Lv F, Wang Z, Sato I, et al. Pathological evidence exploration in deep retinal image diagnosis. In: Proceedings of the AAAI conference on artificial intelligence. vol. 33; 2019. p. 1093-101.

[24]Kinger S, Kulkarni V. Transparent and trustworthy interpretation of COVID-19 features in chest X-rays using explainable AI. Multimedia Tools and Applications. 2025;84(18):19853-81.

[25]Singh D, Brima Y, Levin F, Becker M, Hiller B, Hermann A, et al. An unsupervised XAI framework for dementia detection with context enrichment. Scientific reports. 2025;15(1):39554.

[26]Guo G, Deng L, Tandon A, Endert A, Kwon BC. Mimicri: Towards domain-centered counterfactual explanations of cardiovascular image classification models. In: Proceedings of the 2024 ACM Conference on Fairness, Accountability, and Transparency; 2024. p. 1861-74.

[27]Lamy JB, Sekar B, Guezennec G, Bouaud J, Séroussi B. Explainable artificial intelligence for breast cancer: A visual case-based reasoning approach. Artificial intelligence in medicine. 2019;94:42-53.

[28]van den Brandt A, Christopher M, Zangwill LM, Rezapour J, Bowd C, Baxter SL, et al. GLANCE: Visual Analytics for Monitoring Glaucoma Progression. In: VCBM; 2020. p. 85-96.

[29]Xie Y, Chen M, Kao D, Gao G, Chen X. CheXplain: enabling physicians to explore and understand data-driven, AI-enabled medical imaging analysis. In: Proceedings of the 2020 CHI Conference on Human Factors in Computing Systems; 2020. p. 1-13.

[30]Yang KC, Xu Y, Lin Q, Tang LL, Zhong Jw, An HN, et al. Explainable deep learning algorithm for identifying cerebral venous sinus thrombosis-related hemorrhage (CVST-ICH) from spontaneous intracerebral hemorrhage using computed tomography. EClinicalMedicine. 2025;81.

[31]Giavina-Bianchi M, Vitor WG, Fornasiero de Paiva V, Okita AL, Sousa RM, Machado B. Explainability agreement between dermatologists and five visual explanations techniques in deep neural networks for melanoma AI classification. Frontiers in Medicine. 2023;10:1241484.

[32]Gulum MA, Trombley CM, Ozen M, Esen E, Aksamoglu M, Kantardzic M. Why are explainable AI methods for prostate lesion detection rated poorly by radiologists? Applied Sciences. 2024;14(11):4654.

[33]Gulum MA, Trombley CM, Ozen M, Kantardzic M. Are Post-Hoc Explanation Methods for Prostate Lesion Detection Effective for Radiology End Use? In: 2022 21st IEEE International Conference on Machine Learning and Applications (ICMLA). IEEE; 2022. p. 1183-9.

[34]Hamm CA, Baumgärtner GL, Biessmann F, Beetz NL, Hartenstein A, Savic LJ, et al. Interactive explainable deep learning model informs prostate cancer diagnosis at MRI. Radiology. 2023;307(4):e222276.

[35]Kobayashi K, Takamizawa Y, Miyake M, Ito S, Gu L, Nakatsuka T, et al. Can physician judgment enhance model trustworthiness? A case study on predicting pathological lymph nodes in rectal cancer. Artificial Intelligence in Medicine. 2024;154:102929.

[36]Kumar A, Manikandan R, Kose U, Gupta D, Satapathy SC. Doctor's dilemma: evaluating an explainable subtractive spatial lightweight convolutional neural network for brain tumor diagnosis. ACM Transactions on Multimedia Computing, Communications, and Applications (TOMM). 2021;17(3s):1-26.

[37]Manz R, Baecker J, Cramer S, Meyer P, Müller D, Muzalyova A, et al. Do explainable AI (XAI) methods improve the acceptance of AI in clinical practice? An evaluation of XAI methods on Gleason grading. The Journal of Pathology: Clinical Research. 2025;11(2):e70023.

[38]Karagoz G, van Kollenburg G, Ozcelebi T, Meratnia N. Evaluating How Explainable AI Is Perceived in the Medical Domain: A Human-Centered Quantitative Study of XAI in Chest X-Ray Diagnostics. In: International Workshop on Trustworthy Artificial Intelligence for Healthcare. Springer; 2024. p. 92-108.

[39]Jin W, Fatehi M, Guo R, Hamarneh G. Evaluating the clinical utility of artificial intelligence assistance and its explanation on the glioma grading task. Artificial Intelligence in Medicine. 2024;148:102751.

[40]Trombley CM, Gulum MA, Ozen M, Esen E, Aksamoglu M, Kantardzic M. Evaluating uncertainty-based deep learning explanations for prostate lesion detection. In: Machine Learning for Healthcare Conference. PMLR; 2022. p. 874-91.

[41]Sümer Ö, Huber T, Duong D, Hanchard SEL, Conati C, André E, et al. Evaluation of a Deep Learning and XAI based Facial Phenotyping Tool for Genetic Syndromes: A Clinical User Study. medRxiv. 2025.

[42]Yazdani E, Neizehbaz A, Karamzade-Ziarati N, Kheradpisheh SR. Explainable artificial intelligence for pneumonia classification: clinical insights into deformable prototypical part network in pediatric chest x-ray images. Journal of medical imaging and radiation sciences. 2025;56(5):102023.

[43]Storås AM, Dreyer M, Pahde F, Lapuschkin S, Samek W, Halvorsen P, et al. Exploring the clinical value of concept-based AI explanations in gastrointestinal disease detection. Scientific Reports. 2025;15(1):28860.

[44]Mertes S, Huber T, Weitz K, Heimerl A, André E. Ganterfactual—counterfactual explanations for medical non-experts using generative adversarial learning. Frontiers in artificial intelligence. 2022;5:825565.

[45]Liu J, Zhou X, Lin H, Huang Y, Zheng J, Xu E, et al. Human–machine interaction based on real-time explainable deep learning for higher accurate grading of carotid stenosis from transverse B-mode scan videos. European Journal of Radiology. 2025:112441.

[46]Pham TT, Brecheisen J, Wu CC, Nguyen H, Deng Z, Adjeroh D, et al. ItpCtrl-AI: End-to-end interpretable and controllable artificial intelligence by modeling radiologists' intentions. Artificial intelligence in medicine. 2025;160:103054.

[47]Shi S, Shao Y, Jiang H, Yao Y, Zhang Z, Ding X, et al. MEDebiaser: A Human-AI Feedback System for Mitigating Bias in Multi-label Medical Image Classification. In: Proceedings of the 38th Annual ACM Symposium on User Interface Software and Technology; 2025. p. 1-27.

[48]Marmolejo-Saucedo JA, Kose U. Numerical grad-cam based explainable convolutional neural network for brain tumor diagnosis. Mobile Networks and Applications. 2024;29(1):109-18.

[49]Rezaeian O, Asan O, Bayrak AE. The impact of AI explanations on clinicians' trust and diagnostic accuracy in breast cancer. Applied Ergonomics. 2025;129:104577.

[50]Wysocki O, Mak S, Frost H, Graham DM, Landers D, Aslam T. Translating the machine; An assessment of clinician understanding of ophthalmological artificial intelligence outputs. International journal of medical informatics. 2025;201:105958.

[51]Singh A, Sengupta S, Mohammed AR, Faruq I, Jayakumar V, Zelek J, et al. What is the optimal attribution method for explainable ophthalmic disease classification? In: International Workshop on Ophthalmic Medical Image Analysis. Springer; 2020. p. 21-31.
